# Mosquito species (Diptera, Culicidae) in three ecosystems from the Colombian Andes: identification through DNA barcoding and adult morphology

**DOI:** 10.3897/zookeys.513.9561

**Published:** 2015-07-15

**Authors:** Paula Rozo-Lopez, Ximo Mengual

**Affiliations:** 1 Rheinische Friedrich-Wilhelms-Universität Bonn. An der Immenburg 1, 53121 Bonn, Germany; 2 Zoologisches Forschungsmuseum Alexander Koenig. Adenauerallee 160, 53113 Bonn, Germany

**Keywords:** Culicomorpha, nematocerous Diptera, Neotropical Region, species identification, combining methodologies

## Abstract

Colombia, one of the world’s megadiverse countries, has a highly diverse mosquito fauna and a high prevalence of mosquito-borne diseases. In order to provide relevant information about the diversity and taxonomy of mosquito species in Colombia and to test the usefulness of DNA barcodes, mosquito species collected at different elevations in the departments of Antioquia and Caldas were identified combining adult morphology and barcode sequences. A total of 22 mosquito species from eight genera were identified using these combined techniques. We generated 77 barcode sequences with 16 species submitted as new country records for public databases. We examined the usefulness of DNA barcodes to discriminate mosquito species from the Neotropics by compiling 1,292 sequences from a total of 133 species and using the tree-based methods of neighbor-joining and maximum likelihood. Both methodologies provided similar results by resolving 105 species of mosquitoes separated into distinct clusters. This study shows the importance of combining classic morphological methodologies with molecular tools to accurately identify mosquitoes from Colombia.

## Introduction

Culicidae currently comprises 3,543 formally recognized species distributed throughout most types of habitats and ecosystems of the world (Harbach 2015). Mosquitoes, as a group, occur in a wide array of both aquatic and terrestrial habitats and have correspondingly variable morphological and behavioral adaptations to these (Becker et al. 2010). Due to their hematophagous behavior, mosquitoes are able to transmit many different disease agents such as viruses, bacteria, protozoans, and nematodes from one vertebrate host to another (Becker et al. 2010). The taxonomy of Culicidae has received special attention because of their vector capabilities and medical and veterinary importance (Harbach 2007, Becker et al. 2010). Thus, major mosquito vector species are generally the best studied and understood, both biologically and taxonomically (Harbach 2007).

Mosquito identification is traditionally based on dichotomous keys constructed from morphological features taken for a particular life stage or gender (Munstermann and Conn 1997). The morphological identification of mosquito species is hampered by intraspecific variation, the complexity of some features and the need for specimens in generally excellent condition (Zavortink 1974, Cywinska et al. 2006). The importance of gaining high resolution in mosquito species discrimination requires the implementation of additional tools that can be used for accurate identification (Munstermann and Conn 1997, Cook et al. 2005).

The DNA barcode method was postulated by Hebert et al. (2003a, 2003b) as a DNA sequence-based approach for the accurate identification of specimens and for species discovery. There are three different methods proposed to analyze the efficiency of DNA barcode data to discriminate species: similarity methods based on the match between the query sequence and the reference sequences, tree-based identifications, and the barcode gap (Hebert et al. 2003a, 2004, Ratnasingham and Herbert 2007, Austerlitz et al. 2009). Most published approaches to DNA barcoding use distance-based methods for species designation; however, alternative approaches using character-based phylogenetic analysis have been proposed (DeSalle et al. 2005). Although DNA barcode is now widely use, there are still many open questions about both the advantages and disadvantages of its use, as well as its definition as a methodology (Moritz and Cicero 2004, Brower 2006, Baker et al. 2009, Casiraghi et al. 2010, Jinbo et al. 2011, Wilson et al. 2011, Begsten et al. 2012, Collins and Cruickshank 2013, Stoeckle and Thaler 2014). Despite the known limitations of the method, DNA barcodes have been used to identify specimens and to flag potential new species (e.g. Scheffer et al. 2006, Nagoshi et al. 2011, Tavares et al. 2011, Baldwin and Weigt 2012, Huemer and Mutaten 2015, among others).

In mosquitoes, the effectiveness of the COI barcode marker for specimen identification has been tested in surveys of Canada (Cywinska et al. 2006), India (Kumar et al. 2007), the Iranian islands in the Persian Gulf (Azari-Hamidian et al. 2010), China (Wang et al. 2012), Amazonian Ecuador (Linton et al. 2013), Pakistan (Ashfaq et al. 2014), Singapore (Chan et al. 2014), and Belgium (Versteirt et al. 2015). These studies show correspondence between morphological species and DNA barcode clusters, but also point out the failure of the methodology to distinguish between very similar or cryptic species.

Colombia is located in the northwest of South America. It comprises a variety of biogeographic regions that have contrasting biophysical characteristics and high environmental variability (Etter et al. 2006). Related to this variety of ecosystems, Colombia has high levels of endemism and species richness and has been categorized as a mega-diverse country (Hernandez et al. 1992, Chaves and Arango 1998). Moreover, environmental variability may favor the development and persistence of a great diversity of mosquito species (Foley et al. 2007, Brochero and Quiñones 2008), as well as to favor both the immigration and biological invasion of non-endemic species including vector species (Barreto et al. 1996, Molina et al. 2000, Olano et al. 2001). Furthermore, Colombia is located in a region shown to be a potential hotspot for malaria endemicity and other mosquito borne disease outbreaks (Foley et al. 2008). In Colombia, malaria vector mosquitoes (genus *Anopheles* Meigen, 1818) have been extensively investigated, the morphological keys for their identification are up-to-date and available, and different genetic techniques have been developed to differentiate cryptic species complexes. Nevertheless, the overall diversity of mosquito fauna in Colombia is understudied and generally poorly known (Olano et al. 2001, Montoya-Lerma et al. 2011).

In order to improve the mosquito knowledge in Colombia, we tested the effectiveness of the barcoding methodology to support the reliable identification of Neotropical species of mosquitoes, previously identified with morphological characters, collected over three different altitudinal ecosystems of the Colombian Andes (Antioquia and Caldas departments).

## Materials and methods

### Study area

**Figure 1.**
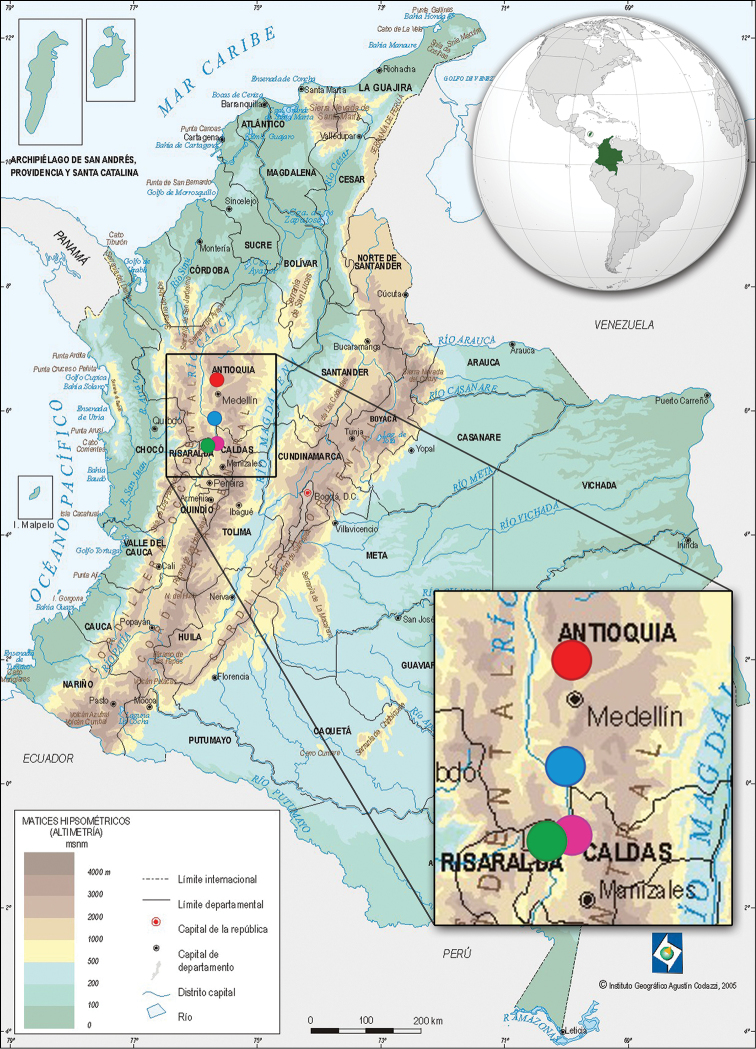
Map of Colombia indicating the sampling sites of mosquitoes collected in this study: Belmira, Antioquia, paramo, 3,200 masl (red circle); Rio Sucio, Caldas, cloud forest, 1,960 masl (green circle); La Pintada, Antioquia, tropical dry forest, 660 masl (blue circle); Supia, Caldas, rural area, 1,150 masl (pink circle). Modified from Instituo Geográfico Agustín Codazzi (www.igac.gov.co) and Wikimedia Commons (by Addicted04).

The study area is located in the west central Andean region in Colombia. Fieldwork was conducted during September 2013 in rural areas of Antioquia and Caldas departments (Fig. 1). This study focused on three important biomes: paramo (Belmira, 3,200 masl), cloud forest (Rio Sucio, 1,960 masl), and tropical dry forest (La Pintada, 660 masl). Each sampling location was split into two habitat types: forest (habitat A) and anthropogenic disturbed area (habitat B). Forest sampling in the paramo plots consisted of dense shrubbery and scattered trees associated with sub-paramo zones. Additional collections were made in the rural area of Supia (Caldas) (mountain forest) at an elevation of 1,150 masl.

### Specimen collection

Mosquito adults were collected using one malaise trap and three Centers for Disease Control-CDC light traps in each habitat type (A or B) of the three biomes, totaling six sites. CDC traps collected for 14 hours, between 5:00 pm and 7:00 am of the next day during two nights in each of our six locations plus the rural area of Supia. Malaise traps were placed and left for 48 hours in each location. Additional specimens were obtained by aspirating mosquitoes attracted to humans during the placement and operation of the traps. All data in the sampled localities were plotted following the protocols of Foley et al. (2010) using a GPS Garmin® Rino 530HCx. Weather data of average wind speed (km/h), temperature (°C), and relative humidity (%) were recorded with a Kestrel® 4000 Weather Tracker.

Wild-caught adults were killed using ethanol (90%) fumes in a lethal chamber to ensure DNA preservation. All mosquitoes were kept dry and individually transferred to labeled 1.5 ml tubes. Each tube was labeled, pierced with a mounted needle (to allow the escape of moisture) and placed in plastic bags containing silica gel. Specimen mounting techniques were conducted using the protocols of Walter Reed Biosystematics Unit-WRBU (Gaffigan and Pecor 1997).

### Adult identification using morphology

All the specimens collected were identified by female morphology and male genitalia. Since there is no single morphological key to facilitate the identification of mosquitoes of Colombia, genus level identifications were made with multiple approaches using the dichotomous keys of Lane (1953), Becker et al. (2010), Chaverri (2009), and the multi-entry web-based keys developed by the WRBU for South America (http://wrbu.si.edu/southcom_MQkeys.html). In addition, available dichotomous keys and original species descriptions were compiled through literature freely available on the WRBU website (www.wrbu.org). Important references used for species identifications by taxonomic groups included: Tribe Aedini (Arnell 1976, Reinert 2000), *Anopheles* (Komp 1942, Cova-Garcia 1961, González and Carrejo 2007), *Culex* Linnaeus, 1758 (Rozeboom and Komp 1950, Cova-Garcia et al. 1966, Bram 1967, Knight and Haeger 1971, Sirivanakarn 1982, Strickmann and Prat 1989, Pecor et al. 1992), *Coquillettidia* Dyar, 1905 (Shannon 1934, Cova-Garcia et al. 1966), *Haemagogus* Williston, 1896 (Kumm et al. 1946, Levi-Castillo 1951, Arnell 1973, Liria and Navarro 2009), *Psorophora* Robineau-Desvoidy, 1827 (Guedes and Souza 1964, Liria and Navarro 2003), *Trichoprosopon* Lane & Cerqueira, 1942 (Stone 1944, Zavortink 1979), *Wyeomyia* Theobald, 1901 (Judd 1998, Motta and de Oliveira 2000). Overall, Lane (1953) was used as a general reference.

### Voucher specimens

Voucher specimens and associated genitalia preparations are stored in the entomological collections of the Zoologisches Forschungsmuseum Alexander Koenig (ZFMK), Bonn (Germany). Genomic DNA extracts are stored at –80 °C in the biobank collections of the ZFMK for future reference. Details of reference material are listed by genus in Suppl. material 1, as well as full collection site details including geo-references and environmental conditions. Fully digitized specimen data records are freely available in MosquitoMap (www.mosquitomap.org). COI barcode sequences can be accessed through GenBank (accession numbers KM592986 to KM593062; see Suppl. material 1).

### Laboratory protocols, DNA extraction, PCR, and sequencing

DNA was extracted from legs and occasionally abdomens (for specimens without legs or with deficient amplification) following standard protocols of the commercially available DNeasy Blood & Tissue Kit (QIAgen®). The COI barcode region was amplified using the forward primer LCO1490 (5’-GGTCAACAAATCATAAAGATATTGG-3’) and the reverse primer C1N2191 (alias Nancy) (5’CCCGGTAAAATTAAAATATAAACTTC-3’) (Simon et al. 1994). When those primers failed to amplify full-length sequences, the set LCO1490 and HCO2198 (5’-TAAACTTCA GGGTGACCAAAAAATCA-3’) (Folmer et al. 1994) were alternatively used. PCR amplification followed the protocol optimized by the Laboratories of Molecular Biology, Alexander Koenig Museum-ZFMK (Bonn, Germany). Each PCR reaction contained 2.5 μL of DNA, 2.3 μL of ddH20, 2 μL of Q-Solution, 10 μL of Qiagen Multiplex-Mix, and 1.6 μM of each primer. The polymerase chain reaction (PCR) cycle conducted was: 95 °C for 15 min, 25 cycles of 94 °C for 35 s, 55 °C for 90 s and 72 °C for 90 s, 72 °C for 10 min and a 10 °C hold. The PCR product was visualized on 1.5% agarose gels, containing 0.5 mg/ml of GelRed®, followed by 1.75 microliters of the PCR product being removed and mixed with 1.75 μL of loading dye. Gels were run at 120 V for 30 min, prior to ultraviolet visualization. PCR products were cleaned using the commercially available QIAquick PCR Purification Kit (QIAgen®). Bi-directionally sequencing reactions were carried out by Macrogen© Inc. Chromatograms were edited in Geneious 7.0.6 (Biomatters© Ltd). Primer sequences were removed from edited contigs and only high-quality sequences of at least 600 bp were retained for data analysis. Sequences were aligned using Geneious 7.0.6.

### Additional sequences

To test variation in the barcoding region across a greater geographical area, barcoding sequences of mosquito species listed for the Neotropics were downloaded from BOLD and GenBank between December 2013 and February 2014 for all identified species with a minimum length of 480 bp of COI barcoding region, no stop codons and alignment without gaps (Suppl. material 2). In order to have a broader representation of mosquito genera, additional sequences of species from other biogeographic areas were downloaded for those Neotropical groups without available sequences. These taxonomic groups include: Coquillettidia (Coquillettidia) Dyar, 1905, Culex (Neoculex) Dyar, 1905, Mansonia (Mansonioides) Theobald, 1907, Ochlerotatus (Protoculex) Felt, 1904, *Orthopodomyia* Theobald, 1904, Toxorhynchites (Lynchiella) Lahille, 1904, and Toxorhynchites (Toxorhynchites) Theobald, 1901. Moreover, an unpublished data set of 45 sequences of mosquitoes collected in rural areas of Uraba (Antioquia, Colombia) during 2009 was also added to the analysis (Suppl. material 3).

*Lutzomyia longipalpis* (Lutz & Neiva, 1912) (Diptera: Psychodidae) was constrained as outgroup. We also included several other genera from three different families as outgroups, i.e. Chironomidae [*Cricotopus bicinctus* (Meigen, 1818), *Chironomus decorus* Goetghebuer, 1927, *Chironomus kiiensis* Tokunaga, 1936, *Dicrotendipes tritomus* (Thienemann & Kieffer, 1916), and *Tanytarsus guerlus* (Roback, 1957)], Simuliidae [*Simulium ochraceum* Walker, 1861, *Simulium inaequale* (Paterson & Shannon, 1927), *Gigantodax abalosi* Wygodzinsky, 1958, and *Gigantodax basinflatus* Wygodzinsky & Coscaron, 1989] and Dixidae [*Dixella* sp.]. A total of 11 outgroup taxa were included. All outgroup taxa sequences were downloaded from BOLD (Suppl. material 4).

### Barcoding methodologies

We used *similarity methods* based on the match between the query sequence and the reference database [e.g. BLAST (http://blast.ncbi.nlm.nih.gov/Blast.cgi) and BOLD Identification System (IDS) (http://www.boldsystems.org/index.php/IDS_OpenIdEngine)] and clustering in *Tree-based identifications* (using Neighbour-joining and maximum likelihood approaches) in order to analyze the DNA barcode region of the mosquitoes collected in Colombia and assign individuals to a given species.

The Neighbour-Joining (NJ) tree (Saitou and Nei 1987) was based on the Tamura-Nei distance model (TN93) (Tamura and Nei 1993) as recommend by Srivathsan and Meier (2012). Bootstrap support values were calculated with 1,000 replicates. The NJ tree, distance matrices and bootstrap were generated using Geneious 7.0.6.

To perform the maximum likelihood analysis, the data set was divided into three partitions according to the codon positions of COI (first, second, and third positions). We determined the best choice of model for each partition using PartionFinder v1.1.0 (Lanfear 2012) under the Akaike Information Criterion (AIC) as recommended by Posada and Buckley (2004). The model chosen for position 1 was SYM+I+G, GTR+I+G for position 2 and GTR+I+G for position 3 of COI gene. Data were analyzed using Garli 2.0 (Zwickl 2006) based on best choice of the model predicted. Default settings (scorethresh-forterm = 0.05; significanttopochange = 0.0001; searchreps = 1) were used to perform 20 replicates. The tree with the highest likelihood was retained. Bootstrap support values were estimated from 1,000 replicates using the same independent models. Analytical runs of ML were performed at the Hydra cluster (Center for Astrophysics, Harvard University), a Linux based cluster running Grid Engine with more than 3,000 CPUs with AMD 64-bit processors.

## Results

A total of 77 mosquito specimens were collected from four sampling sites during our study (Table 1). Sampling success among traps varies greatly across location and habitat types. Belmira, the location with highest wind speed and precipitation, only reported one or no specimens for their two habitats. The specimens were identified to 22 species from eight genera. One species collected during this study in La Pintada forest habitat, *Wyeomyia luteoventralis* Theobald, 1901, is the first record for Colombia (Rozo-Lopez and Mengual, 2015).

**Table 1.** Mosquito species collected. Four collection sites of the Colombian Andes during September 2013. One specimen collected in Rio Sucio (habitat A) still remains undetermined due to damage and lack of conspecific sequences for comparison.

Mosquito species	Sites						
	Supia 1,150 masl	Rio Sucio 1,960 masl		La Pintada 660 masl		Belmira 3,200 masl	
Species		Habitat A	Habitat B	Habitat A	Habitat B	Habitat A	Habitat B
*Anopheles neomaculipalpus*	1						
*Coquillettidia nigricans*					1		
*Culex coniger*		10					
*Culex conspirator*				5			
*Culex* spp. [*coronator* complex]		1	1	3			
*Culex declarator*	1			10			
*Culex educator*				1			
*Culex erraticus*				6	2		
*Culex erythrothorax*		1					
*Culex lactator*				3			
*Culex lucifugus*				6			
*Culex nigripalpus*	1			2			
*Culex spinosus*		1					
*Culex spissipes*					1		
*Culex theobaldi*				1			
Culex (Culex) sp.		1					
Culex (Melanoconion) sp.				4	1		
*Haemagogus janthinomys*					2		
*Haemagogus lucifer*					1		
*Ochlerotatus angustivittatus*	1			1			
*Ochlerotatus euiris*						1	
*Psorophora cingulata*					1		
*Psorophora ferox*				2	1		
*Trichoprosopon evansae*		1					
*Wyeomyia luteoventralis*				1			
Undetermined		1					
**Total**	**4**	**16**	**1**	**45**	**10**	**1**	**0**

### Adult identification using morphology

Twenty one species belonging to seven genera were successfully identified by morphological characteristics of adult females and male genitalia: *Coquillettidia nigricans* Coquillett, 1904 [n=1 female], *Culex conspirator* Dyar & Knab, 1906 [n=5 males], *Culex corniger* Theobald, 1903 [n=8 females], *Culex declarator* Dyar & Knab, 1906 [n=8; 5 females, 3 males], *Culex educator* Dyar & Knab, 1906 [n=1 male], *Culex erraticus* [n=2 males], *Culex erythrothorax* Dyar, 1907 [n=1 female], *Culex lactator* Dyar & Knab, 1906 [n=2 females], *Culex lucifugus* Komp, 1936 [n=3 males], *Culex nigripalpus* Theobald, 1901 [n=2; 1 female, 1 male], *Culex spinosus* Lutz, 1905 [n=1 male], *Culex spissipes* Theobald, 1903 [n=1 female], *Culex theobaldi* Lutz, 1094 [n=1 male], *Haemagogus janthinomys* Dyar, 1921 [n=2 females], *Haemagogus lucifer* Howard, Dyar & Knab, 1913 [n=1 female], *Ochlerotatus angustivittatus* Dyar & Knab, 1907 [n=2; 1 female, 1 male], *Ochlerotatus euiris* Dyar, 1922 [n=1 female], *Psorophora cingulata* Fabricius, 1805 [n=1 female], *Psorophora ferox* von Humboldt, 1819 [n=3; 2 females, 1 male], *Trichoprosopon evansae* Antunes, 1942 [n=1 female], and *Wyeomyia luteoventralis* [n=1 female]. One specimen of the genus *Anopheles* was identified by morphology to the group: *neomaculipalpus* / *punctimacula*. It was only possible to identify the species using its gene sequence.

### Mosquito barcoding

All the collected specimens were successfully amplified and sequenced, generating a total of 77 sequences with lengths ranging from 618 to 699 bp. Only high quality sequences were retained. Sequences from 14 species are submitted as new records for public databases: *Coquillettidia nigricans*, *Culex conspirator*, *Culex educator*, *Culex lactator*, *Culex lucifugus*, *Culex spissipes*, *Culex theobaldi*, *Haemagogus janthinomys*, *Haemagogus lucifer*, *Ochlerotatus angustivittatus*, *Ochlerotatus euiris*, *Psorophora cingulata*, *Trichoprosopon evansae*, *Wyeomyia luteoventralis*.

All the DNA sequences obtained were compared to those available, by 25 January 2015, in GenBank and BOLD Identification System. At the level of genus, BLAST accurately discriminated 92% of the genera previously identified, whereas BOLD accurately discriminated 70% of genera previously identified. Although BLAST represented a higher percentage of accurate discrimination of genera in our queries, five specimens with a score of more than 89% were wrongly matched: *Trichoprosopon* was mismatched as *Anopheles*, *Wyeomyia* was mismatched as *Sabethes* Robineau-Desvoidy, 1827, *Haemagogus* was mismatched as *Ochlerotatus* (or *Aedes*), and for the sequence of *Haemagogus lucifer* mixed results of *Spilogona* Schnabl, 1911, *Haematobia* Lepeletier & Serville, 1828 (family Muscidae), and *Culex* were obtained.

By adding GenBank, BOLD, and unpublished sequences to our data set, it was possible to obtain taxon coverage of 68% of the mosquito genera and 34% of the mosquito species listed for Colombia. Moreover, our data set coverage for Neotropical mosquito species corresponds to 58% of mosquito genera and 12% of mosquito species. The data set of the COI sequences of the Neotropical mosquitoes comprises a total of 1,292 barcode sequences belonging to 133 species and 21 genera (with a minimum length of 640 bp). The alignment was unambiguous: no gaps and amino acid translations without stop codons, indicating that all sequences represented functional protein coding genes, not pseudogenes. The analyzed region starts at the position 45 and stops at position 693 of the COI gene of the mitochondrial genome of *Drosophila melanogaster* Meigen, 1830 (AJ400907) (Adams et al. 2000) used as the reference genome. The amino acid reading frame starts at the second base of the dataset. The data matrix shows 116 invariant sites and a mean A+T content of 67.4%.

The Neighbour-Joining and Maximum likelihood analyses showed 105 species, from the 133 Neotropical species with available barcodes, separated into distinct clusters. Genera represented by more than one taxon formed cohesive assemblages of five clusters [*Anopheles*, *Orthopodomyia*, *Psorophora*, *Toxorhynchites* Theobald, 1901, *Uranotaenia* Lynch Arribalzaga, 1891] in the NJ analysis and eight [*Anopheles*, *Coquillettidia*, *Culex*, *Orthopodomyia*, *Psorophora*, *Stegomyia* Theobald, 1901, *Toxorhynchites*, *Uranotaenia*] in the ML analysis.

**Figure 2.**
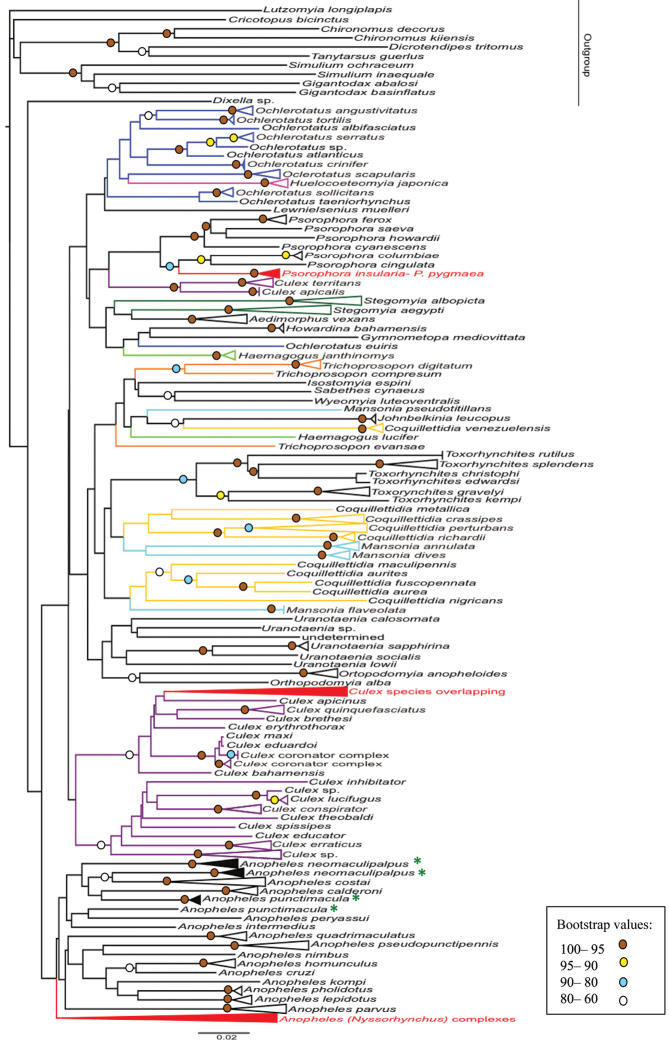
Neighbour-Joining tree of the barcoding sequences of mosquito species listed for Neotropics, based on Tamura-Nei genetic distances. Terminal branches have been collapsed in order to save space (see original tree in Suppl. material 5 and 6). Branches in colors indicate non-monophyletic genera. Red clusters represent groups with problems to discriminate species. Names with green asterisk indicate non- monophyletic species. Bootstrap values above 60 (1,000 replicates) are given at the nodes.

The NJ tree based on Tamura-Nei genetic distances (Fig. 2) revealed most of the species clusters (100 species) with high bootstrap value (97–99). Conversely, 28 species were recovered as non-monophyletic groups during this analysis: *Anopheles punctimacula* Dyar & Knab, 1906 and *Anopheles neomaculipalpus* Curry, 1931 as paraphyletic groups, an overlapping of *Psorophora insularia* (Dyar & Knab, 1906) and *Psorophora pygmaea* (Theobald, 1903), four species of *Culex* [*Culex declarator*, *Culex nigripalpus*, *Culex conspirator*, and *Culex spinosus*], and 20 species of Anopheles (Nyssorynchus) Blanchard, 1902 [*myzorynchella* section = *Anopheles antunesi* Galvao & Amarai, 1940, *Anopheles lutzii* Cruz, 1901, *Anopheles pristinus* Nagaki & Sallum, 2010; *nuneztovari* complex = *Anopheles goeldi* Rozeboom & Gabaldon, 1941, *Anopheles dunhami*, *Anopheles nuneztovari* Gabaldon, 1940; *oswaldoi* complex = *Anopheles evansae* (Brethes, 1926), *Anopheles galvaoi* Causey, Deane & Deane, 1943, *Anopheles konderi* Galvao & Damasceno, 1942, *Anopheles oswaldoi* (Peryassu, 1922), *Anopheles rangeli* Gabaldon, Cova-Garcia & Lopez, 1940; *strodei* complex = *Anopheles albertoi* Unti, 1941, *Anopheles arthuri* Unti, 1941, *Anopheles rondoni* (Neiva & Pinto, 1922), *Anopheles strodei* Root, 1926; *albitarsis* complex = *Anopheles albitarsis* Lynch Arribalzaga, 1878, *Anopheles janconnae* Wilkerson & Sallum, 2009, *Anopheles marajoara* Galvao & Damasceno, 1942, *Anopheles oryzalimnetes* Wilkerson & Motoki, 2009; and *Anopheles benarrochi* Gabaldon, Cova-Garcia & Lopez, 1941].

**Figure 3.**
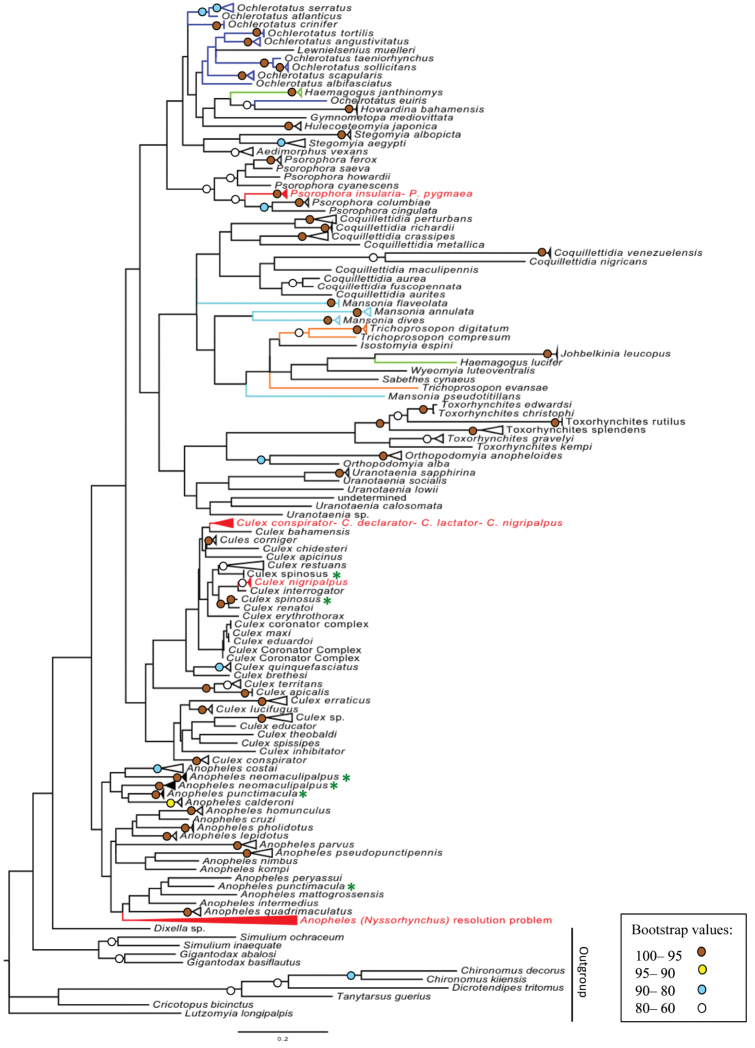
Maximum likelihood tree of the barcoding sequences of mosquito species listed for the Neotropics. Terminal branches have been collapsed in order to save space (see original tree in Suppl. material 7 and 8). Branches in colors indicate non-monophyletic genera. Red clusters represent groups with problems to discriminate species. Names with green asterisk indicate non-monophyletic species. Bootstrap values above 60 (1,000 replicates) are given at the nodes.

The likelihood score for the best ML tree was –31,760.30891. The overall topologies of the ML (Fig. 3) and NJ trees compared favorably with exception of *Coquillettidia*, *Culex*, and *Stegomyia*, which resolved as monophyletic clusters. The ML tree revealed 92 species clusters with high bootstrap (95–99). The bootstrap on the remaining 13 species ranged from 65 to 93. ML analysis also recovered 28 species as non-monophyletic groups.

## Discussion

In this study 22 species belonging to eight genera and 11 subgenera were identified by combining morphological and molecular methodologies. Although our data and sampling size were limited, the combination of methodologies provided better success in species identification. Overall, congruence between morphology and barcode grouping, based on cluster monophyly with high (more than 95%) bootstrap support, was found in 18 of the 22 morphologically defined taxa (82%). Similarity methods based on the match between the query sequence and the reference database (more than 98% identity between BOLD/BLAST) only discriminated three mosquito species of this study. Similarity methods based on the match between the query sequence and the reference databases represent the least suitable method to discriminate mosquito species in this study.

Tree-based diagnostics are graphic criterion for species recognition, which describes genetic similarity in a visually satisfying style (Goldstein and DeSalle 2010). An important advantage of using a tree-based approach is that they present a direct sense of the statistical reliability and do not retrieve a positive result if no matching diagnostics are found (unlike the best match algorithm of BLAST and BOLD which retrieves the closest match but requires the user to interpret its reliability) (Goldstein and DeSalle 2010). Here, we compared two tree-based methods (neighbour-joining and maximum likelihood), which provided similar results. The NJ tree computed was in general agreement with previously published taxonomy based approaches (Hebert et al. 2003a, 2003b, Hebert et al. 2004, Hajibabaei et al. 2007, Cywinska et al. 2006, Kumar et al. 2007). Both tree-based analysis in this study showed 105 species of mosquitoes (from 133 total species) separated into distinct clusters. Since the NJ clustering performed considerably faster than ML approach and indeed has been used in the great majority of published barcoding studies, these results indicate it is an efficient choice for mosquito barcode analyses, similar to what is observed for other organisms (Little and Stevenson 2007, Elias et al. 2007).

The usefulness of DNA barcodes to discriminate mosquito genera of our dataset by using a tree-based approach was poorly supported. Approximately half of currently recognized genera represented by two or more species formed stable clusters in the NJ tree. Similarly, generic monophyly was weakly improved with the ML approach. In other insect genera, the monophyletic clusters based on DNA barcodes varies greatly depending on clustering method: e.g. Lepidoptera: Ithomiinae 50 to 61% recovered generic monophyly (Elias et al. 2007), Diptera: Chironomidae 40% (Ekrem et al. 2007, 2010), Simuliidae 62% (Rivera and Currie 2009) and Muscidae 40% (Renaud et al. 2012), Hymenoptera: Apoidea 100% success (Sheffield et al. 2009). It remains unclear whether this is due to lack of phylogenetic signal in COI at this depth, the type of tree-building method, or to the true lack of monophyly of genera as currently defined (Renaud et al. 2012). Conversely, a high level of correspondence at the species level is observed between morphology and molecular species limits in the tree-based approach in the present study. The performance of DNA-based specimen identification in Diptera using COI differs greatly in the literature, which varies from less than 50% to near 100% congruence levels (Cywinska et al. 2006, Smith et al. 2006, Whitworth et al. 2007, Rivera and Currie 2009, Meiklejohn et al. 2012, Renaud et al. 2012, Smit et al. 2013, Contrearas et al. 2014). In most of the studies, identification success rose upon relaxing the bootstrap requirement.

In tree-based methods, the non-monophyly at the species level represents the greatest challenge for taxon sampling and threshold approach (Meyer and Paulay 2005). Funk and Omland (2003) explained five possible reasons for non-monophyly at the species level, i.e. inadequate phylogenetic information, imperfect taxonomy, interspecific hybridization, incomplete lineage sorting, and unrecognized paralogy. There are cases in which tree-based analyses of DNA barcodes have failed to discriminate species of insects (Wiemers and Fiedler 2007, Whitworth et al. 2007, Foster et al. 2013). The non-monophyletic groups of our study includes thirty species of *Anopheles*, ﻿two species of *Psorophora*, ﻿and four or five, depending on the tree reconstruction method, species of *Culex*.

*Culex* was the most diverse and species rich genus in the sampled mosquitoes. *Culex* is a cosmopolitan genus and one of the largest groups of the family Culicidae (768 species divided among 26 subgenera). There are many areas of uncertainty regarding phylogenetic relationships within the genus, as well as some problems with the identification of some species. In our study, many difficulties arose attempting to identify specimens of *Culex* species. In many species, female identification is very problematic due to polymorphisms and ambiguities of the morphological characters (Forattini 2002, Laurito et al. 2013). Morphological identification of *Culex* species is based primarily on differences in male genitalia (Harbach 2011). Moreover, the presence of unknown species complexes within *Culex* makes species identification challenging (Harbach 2011, Laurito et al. 2013). As research previously suggested, the non-monophyly of given species of *Culex* approached during this study is not a surprise. Furthermore, in earlier studies only 42% of the Culex (Culex) previously identified morphologically to species were clustered with their conspecifics in the NJ tree (Laurito et al. 2013). Although our data are limited, present results point in the same direction and more taxonomic work is needed to assess the monophyly of *Culex* and the phylogenetic relationships within the genus.

Classification of the species within the genus *Anopheles* has always been challenging. The current system of subgeneric classification is based primarily on characteristics of the male genitalia (Harbach 2013). Many *Anopheles* species are morphologically indistinguishable and form cryptic species complexes, which require a molecular approach as the only effective tool for resolving their identification (Foster et al. 2013). The DNA barcode approach has been successfully used in corroborating lineages within the *Anopheles albitarsis* complex (Ruiz-Lopez et al. 2012). In the present study, difficulties in resolving species among the sequences of the subgenus Anopheles (Nyssorhynchus)﻿ were expected. Most of the overlapping of species was obtained among one section (*myzorynchella* section) and four of the five complexes comprising this group (*nuneztovari*, *oswaldoi*, *strodei*, *albitarsis*). Even if species level could not be reached for this difficult subgenus, barcodes allowed us to clearly identify the different species complexes present. Single genes, including the COI barcode region, are poor at confirming morphologically defined species and to estimate phylogenetic relationships within the subgenus *Nyssorhynchus* (Foster et al. 2013). However, a multi-locus approach (COI barcode region, nuclear white and CAD genes) was able to discriminate *Nyssorhynchus* species with greater accuracy (Foster et al. 2013).

The most important factor affecting the accuracy of species identification through public databases is the coverage and reliability of available sequences (Ekrem et al. 2007, Wiemers and Fiedler 2007, Virgilio et al. 2010). Unfortunately, the GenBank and BOLD databases have many records believed to be from misidentified specimens (Harris 2003, Meier and Dikow 2004, Meier et al. 2008), which are only obvious by incorporating accurately identified specimens into databases (Meyer and Paulay 2005, Meier et al. 2006). The importance of voucher specimens which can be reexamined is paramount. Furthermore, erroneous sequences may lead to an underestimation of the potential for species discrimination by DNA barcodes (Pape et al. 2009, Collins and Cruickshank 2013). In total, sequences for 133 Neotropical species were included in the tree-based analysis, which displayed species discrimination among most of the taxa, but not for all. We expect that as more studies provide sampling data, the number of entries will increase in databases, which should greatly improve the accuracy of search queries. Nonetheless, results of best match retrieved from any of the databases should not be used indiscriminately without considering the reliability of the comparison. Although BLAST and BOLD Identification System retrieved incongruent identifications for some of our sequences, these search tools are useful to check for sequence contaminations, especially when a species is sequenced for first time.

It is clear that reference libraries with properly identified sequences will facilitate the association of conspecific specimens and the detection of identification errors. The DNA barcodes produced in this work allowed for the identification of females and damaged specimens, which could not be done using morphological characteristics alone. This study highlights the potential of barcoding methodology to resolve taxonomic problems associated with limitations in morphological identification, but also draws attention to its limitations to discriminate species in some Neotropical mosquito genera, especially in Anopheles (Nyssorhynchus) ﻿species complexes and some *Culex* species. A larger taxon barcode library with correctly identified vouchers will help future studies. Despite the limitations in our survey, the DNA barcodes produced in this work are an important contribution to increase the scope of reference libraries with properly identified sequences and to assist in the identification of mosquito species from Colombia.

## Supplementary materials

Supplementary material 1 Mosquito specimens collected in the present study Data type: occurrence Explanation note: Full collection site details, including geo-references and environmental conditions, of the mosquito taxa identified in this study. This dataset is made available under the Open Database License (http://opendatacommons.org/licenses/odbl/1.0/). The Open Database License (ODbL) is a license agreement intended to allow users to freely share, modify, and use this Dataset while maintaining this same freedom for others, provided that the original source and author(s) are credited.Paula Rozo-Lopez, Ximo Mengual

Supplementary material 2 COI mosquito sequences downloaded from NCBI and BOLD Data type: Text Explanation note: Sequences from NCBI and BOLD. Sequences downloaded between December 2013 and February 2014 for all identified species with a minimum length of 480 bp of COI barcoding region, no stop codons and possible alignment among the majority of the sequences. Total of 1,159 sequences. In blue: Additional species sequences from other geographic areas for those Neotropical groups without available sequences from the Neotropics. This dataset is made available under the Open Database License (http://opendatacommons.org/licenses/odbl/1.0/). The Open Database License (ODbL) is a license agreement intended to allow users to freely share, modify, and use this Dataset while maintaining this same freedom for others, provided that the original source and author(s) are credited.Paula Rozo-Lopez, Ximo Mengual

Supplementary material 3 Mosquito specimens collected in Uraba during 2009 (unpublish data) Data type: occurrence Explanation note: Additional sequences of mosquitoes collected in rural areas of Uraba (Antioquia, Colombia) during 2009, including sampling information. This dataset is made available under the Open Database License (http://opendatacommons.org/licenses/odbl/1.0/). The Open Database License (ODbL) is a license agreement intended to allow users to freely share, modify, and use this Dataset while maintaining this same freedom for others, provided that the original source and author(s) are credited.Paula Rozo-Lopez, Ximo Mengual

Supplementary material 4 Outgroup taxa used in the present study Data type: Text Explanation note: Outgroup taxa used in the present study. Sequences downloaded from BOLD between December 2013 and February 2014. This dataset is made available under the Open Database License (http://opendatacommons.org/licenses/odbl/1.0/). The Open Database License (ODbL) is a license agreement intended to allow users to freely share, modify, and use this Dataset while maintaining this same freedom for others, provided that the original source and author(s) are credited.Paula Rozo-Lopez, Ximo Mengual

Supplementary material 5 NJ tree based on 1,292 sequences of Neotropical mosquitoes Data type: picture Explanation note: Original Neighbour-Joining tree of the barcoding sequences of mosquito species listed for the Neotropics, based on Tamura-Nei genetic distances. This dataset is made available under the Open Database License (http://opendatacommons.org/licenses/odbl/1.0/). The Open Database License (ODbL) is a license agreement intended to allow users to freely share, modify, and use this Dataset while maintaining this same freedom for others, provided that the original source and author(s) are credited.Paula Rozo-Lopez, Ximo Mengual

Supplementary material 6 NJ tree based on 1,292 sequences of Neotropical mosquitoes Data type: multimedia Explanation note: Original Neighbour-Joining tree of the barcoding sequences of mosquito species listed for the Neotropics, based on Tamura-Nei genetic distances (.tre). This dataset is made available under the Open Database License (http://opendatacommons.org/licenses/odbl/1.0/). The Open Database License (ODbL) is a license agreement intended to allow users to freely share, modify, and use this Dataset while maintaining this same freedom for others, provided that the original source and author(s) are credited.Paula Rozo-Lopez, Ximo Mengual

Supplementary material 7 ML tree based on 1,292 sequences of Neotropical mosquitoes Data type: picture Explanation note: Original Maximum likelihood tree of the barcoding sequences of mosquito species listed for the Neotropics. This dataset is made available under the Open Database License (http://opendatacommons.org/licenses/odbl/1.0/). The Open Database License (ODbL) is a license agreement intended to allow users to freely share, modify, and use this Dataset while maintaining this same freedom for others, provided that the original source and author(s) are credited.Paula Rozo-Lopez, Ximo Mengual

Supplementary material 8 ML tree based on 1,292 sequences of Neotropical mosquitoes Data type: multimedia Explanation note: Original Maximum likelihood tree of the barcoding sequences of mosquito species listed for the Neotropics (.tre). This dataset is made available under the Open Database License (http://opendatacommons.org/licenses/odbl/1.0/). The Open Database License (ODbL) is a license agreement intended to allow users to freely share, modify, and use this Dataset while maintaining this same freedom for others, provided that the original source and author(s) are credited.Paula Rozo-Lopez, Ximo Mengual
