# Supplementary material for: Mosquito species (Diptera, Culicidae) in three ecosystems from the Colombian Andes: identification through DNA barcoding and adult morphology
Source: Zookeys. 2015 Jul 15;(513):39–64. doi: 10.3897/zookeys.513.9561 (PMC4524277; doi:10.3897/zookeys.513.9561)
Supplement: Supplementary material 1 — Mosquito specimens collected in the present study [file zookeys-513-039-s001.docx]

**APPENDIX 1**

Mosquito taxa identified in this study, their gender, GenBank information and full collection site details, including geo-references and environmental conditions.

| **Species** | **Gender** | **GenBank accession number** | **Code** | **Label information** | **Habitat aspects** | **Weather conditions** |
| --- | --- | --- | --- | --- | --- | --- |
| *Anopheles (Anopheles) neomaculipalpus* | Female | KM592986 | ZFMK-PR001 | COLOMBIA: Caldas Dpto., Supia. N5.43780º W75.64794º. 1150m. 28.ix. 2013. CDC | Rural area |  |
| *Coquillettidia (Rhyncotaenia) nigricans* | Female | KM593033 | ZFMK-PR048 | COLOMBIA: Antioquia Dpto, La Pintada. N05.63622º W075.58782º. 659m. 24.ix. 2013, CDC. | Tropical dry forest- Disturbed | 30 °C; 70.9 %R.H.; 0 km/h Average wind Speed; 0 km/h max. wind Speed |
| *Culex (Culex)* c.f. *erythrothorax* | Female | KM593011 | ZFMK-PR026 | COLOMBIA: Caldas Dpto, Rio Sucio. N05.40523º W075.70572º. 1978m., 28.ix. 2013, CDC. | Cloud forest- Forest | 23.8 °C; 77.1 %R.H.; 0 km/h Average wind Speed; 0 km/h max. wind Speed |
| *Culex (Culex) declarator* | Female | KM592993 | ZFMK-PR008 | COLOMBIA: Antioquia Dpto, La Pintada, N05.63646º W075.59002º. 710m. 25.ix. 2013, CDC. | Tropical dry forest- Forest | 28.3 °C; 94.8 %R.H.; 0 km/h Average wind Speed; 0 km/h max. wind Speed |
| *Culex (Culex) declarator* | Female | KM592995 | ZFMK-PR010 | COLOMBIA: Antioquia Dpto, La Pintada, N05.63646º W075.59002º. 710m. 24.ix. 2013, CDC. | Tropical dry forest- Forest | 27.3 °C; 80.4 %R.H.; 0 km/h Average wind Speed; 0 km/h max. wind Speed |
| *Culex (Culex) declarator* | Female | KM593007 | ZFMK-PR022 | COLOMBIA: Antioquia Dpto, La Pintada, N05.63646º W075.59002º. 710m. 24.ix. 2013, CDC. | Tropical dry forest- Forest | 27.3 °C; 80.4 %R.H.; 0 km/h Average wind Speed; 0 km/h max. wind Speed |
| *Culex (Culex) declarator* | Female | KM593010 | ZFMK-PR025 | COLOMBIA: Caldas Dpto., Supia. N5.43780º W75.64794º. 1150m. 28.ix. 2013. CDC | Rural area |  |
| *Culex (Culex) declarator* | Female | KM593014 | ZFMK-PR029 | COLOMBIA: Antioquia Dpto, La Pintada, N05.63646º W075.59002º. 710m. 25.ix.2013, CDC. | Tropical dry forest- Forest | 28.3 °C; 94.8 %R.H.; 0 km/h Average wind Speed; 0 km/h max. wind Speed |
| *Culex (Culex) declarator* | Female | KM593017 | ZFMK-PR032 | COLOMBIA: Antioquia Dpto, La Pintada, N05.63646º W075.59002º. 710m. 25.ix. 2013, CDC. | Tropical dry forest- Forest | 28.3 °C; 94.8 %R.H.; 0 km/h Average wind Speed; 0 km/h max. wind Speed |
| *Culex (Culex) declarator* | Female | KM593019 | ZFMK-PR034 | COLOMBIA: Antioquia Dpto, La Pintada, N05.63646º W075.59002º. 710m. 25.ix. 2013, CDC. | Tropical dry forest- Forest | 28.3 °C; 94.8 %R.H.; 0 km/h Average wind Speed; 0 km/h max. wind Speed |
| *Culex (Culex) declarator* | Male | KM593046 | ZFMK-PR061 | COLOMBIA: Antioquia Dpto, La Pintada, N05.63646º W075.59002º. 710m. 24.ix. 2013, CDC. | Tropical dry forest- Forest | 27.3 °C; 80.4 %R.H.; 0 km/h Average wind Speed; 0 km/h max. wind Speed |
| *Culex (Culex) declarator* | Male | KM593051 | ZFMK-PR066 | COLOMBIA: Antioquia Dpto, La Pintada, N05.63646º W075.59002º. 710m. 24.ix. 2013, CDC. | Tropical dry forest- Forest | 27.3 °C; 80.4 %R.H.; 0 km/h Average wind Speed; 0 km/h max. wind Speed |
| *Culex (Culex) declarator* | Male | KM593055 | ZFMK-PR070 | COLOMBIA: Antioquia Dpto, La Pintada, N05.63646º W075.59002º. 710m. 24.ix. 2013, CDC. | Tropical dry forest- Forest | 27.3 °C; 80.4 %R.H.; 0 km/h Average wind Speed; 0 km/h max. wind Speed |
| *Culex (Culex) declarator* | Male | KM593057 | ZFMK-PR072 | COLOMBIA: Antioquia Dpto, La Pintada, N05.63646º W075.59002º. 710m. 25.ix. 2013, CDC. | Tropical dry forest- Forest | 28.3 °C; 94.8 %R.H.; 0 km/h Average wind Speed; 0 km/h max. wind Speed |
| *Culex (Culex) nigripalpus* | Female | KM592991 | ZFMK-PR006 | COLOMBIA: Antioquia Dpto, La Pintada, N05.63646º W075.59002º. 710m. 25.ix. 2013, CDC. | Tropical dry forest- Forest | 28.3 °C; 94.8 %R.H.; 0 km/h Average wind Speed; 0 km/h max. wind Speed |
| *Culex (Culex) nigripalpus* | Female | KM592992 | ZFMK-PR007 | COLOMBIA: Antioquia Dpto, La Pintada, N05.63646º W075.59002º. 710m. 25.ix. 2013, CDC. | Tropical dry forest- Forest | 28.3 °C; 94.8 %R.H.; 0 km/h Average wind Speed; 0 km/h max. wind Speed |
| *Culex (Culex) nigripalpus* | Female | KM593008 | ZFMK-PR023 | COLOMBIA: Caldas Dpto, Supia. N5.43780º W75.64794º. 1150m. 28.ix. 2013. CDC | Rural area |  |
| *Culex (Culex) nigripalpus* | Male | KM593058 | ZFMK-PR073 | COLOMBIA: Antioquia Dpto, La Pintada, N05.63646º W075.59002º. 710m. 25.ix. 2013, CDC. | Tropical dry forest- Forest | 28.3 °C; 94.8 %R.H.; 0 km/h Average wind Speed; 0 km/h max. wind Speed |
| *Culex (Culex)* sp. [*coronator* complex] | Female | KM592996 | ZFMK-PR011 | COLOMBIA: Antioquia Dpto, La Pintada, N05.63646º W075.59002º. 710m. 25.ix. 2013, CDC. | Tropical dry forest- Forest | 28.3 °C; 94.8 %R.H.; 0 km/h Average wind Speed; 0 km/h max. wind Speed |
| *Culex (Culex)* sp. [*coronator* complex] | Female | KM593009 | ZFMK-PR024 | COLOMBIA: Caldas Dpto, Rio Sucio. N05.40523º W075.70572º. 1978m. 28.ix. 2013, CDC. | Cloud forest- Forest | 23.8 °C; 77.1 %R.H.; 0 km/h Average wind Speed; 0 km/h max. wind Speed |
| *Culex (Culex)* sp. [*coronator* complex] | Female | KM593013 | ZFMK-PR028 | COLOMBIA: Caldas Dpto, Rio Sucio. N05.41012º W075.71290º. 1958m. 28.ix. 2013, CDC. | Cloud forest- Disturbed | 20.5 °C; 87.3 %R.H.; 1.7 km/h Average wind Speed; 3.1 km/h max. wind Speed |
| *Culex (Culex)* sp. [*coronator* complex] | Female | KM593016 | ZFMK-PR031 | COLOMBIA: Antioquia Dpto, La Pintada, N05.63646º W075.59002º. 710m. 24.ix. 2013, CDC. | Tropical dry forest- Forest | 27.3 °C; 80.4 %R.H.; 0 km/h Average wind Speed; 0 km/h max. wind Speed |
| *Culex (Culex)* sp. [*coronator* complex] | Female | KM593018 | ZFMK-PR033 | COLOMBIA: Antioquia Dpto, La Pintada, N05.63646º W075.59002º. 710m. 25.ix. 2013, CDC. | Tropical dry forest- Forest | 28.3 °C; 94.8 %R.H.; 0 km/h Average wind Speed; 0 km/h max. wind Speed |
| *Culex (Culex)* sp. | Male | KM593060 | ZFMK-PR075 | COLOMBIA: Caldas Dpto, Rio Sucio. N05.40523º W075.70572º. 1978m. 27.ix. 2013, CDC. | Cloud forest- Forest | 19.3 °C; 89.4 %R.H.; 0 km/h Average wind Speed; 0 km/h max. wind Speed |
| *Culex (Culex) spinosus* | Male | KM593059 | ZFMK-PR074 | COLOMBIA: Caldas Dpto, Rio Sucio. N05.40523º W075.70572º. 1978m. 27.ix. 2013, CDC. | Cloud forest- Forest | 19.3 °C; 89.4 %R.H.; 0 km/h Average wind Speed; 0 km/h max. wind Speed |
| *Culex (Melanoconion)* c.f. *spissipes* | Female | KM593030 | ZFMK-PR045 | COLOMBIA: Antioquia Dpto, La Pintada. N05.63622º W075.58782º. 659m. 25.ix. 2013, CDC. | Tropical dry forest- Disturbed | 37.2 °C; 47 %R.H.; 4.6 km/h Average wind Speed; 4.7 km/h max. wind Speed |
| *Culex (Melanoconion) conspirator* | Male | KM593043 | ZFMK-PR058 | COLOMBIA: Antioquia Dpto, La Pintada, N05.63646º W075.59002º. 710m. 24.ix. 2013, CDC. | Tropical dry forest- Forest | 27.3 °C; 80.4 %R.H.; 0 km/h Average wind Speed; 0 km/h max. wind Speed |
| *Culex (Melanoconion) conspirator* | Male | KM593048 | ZFMK-PR063 | COLOMBIA: Antioquia Dpto, La Pintada, N05.63646º W075.59002º. 710m. 24.ix. 2013, CDC. | Tropical dry forest- Forest | 27.3 °C; 80.4 %R.H.; 0 km/h Average wind Speed; 0 km/h max. wind Speed |
| *Culex (Melanoconion) conspirator* | Male | KM593050 | ZFMK-PR065 | COLOMBIA: Antioquia Dpto, La Pintada, N05.63646º W075.59002º. 710m. 24.ix. 2013, CDC. | Tropical dry forest- Forest | 27.3 °C; 80.4 %R.H.; 0 km/h Average wind Speed; 0 km/h max. wind Speed |
| *Culex (Melanoconion) conspirator* | Male | KM593053 | ZFMK-PR068 | COLOMBIA: Antioquia Dpto, La Pintada, N05.63646º W075.59002º. 710m. 24.ix. 2013, CDC. | Tropical dry forest- Forest | 27.3 °C; 80.4 %R.H.; 0 km/h Average wind Speed; 0 km/h max. wind Speed |
| *Culex (Melanoconion) conspirator* | Male | KM593054 | ZFMK-PR069 | COLOMBIA: Antioquia Dpto, La Pintada, N05.63646º W075.59002º. 710m. 24.ix. 2013, CDC. | Tropical dry forest- Forest | 27.3 °C; 80.4 %R.H.; 0 km/h Average wind Speed; 0 km/h max. wind Speed |
| *Culex (Melanoconion) educator* | Male | KM593042 | ZFMK-PR057 | COLOMBIA: Antioquia Dpto, La Pintada. N05.63622º W075.58782º. 659m. 24.ix. 2013, CDC. | Tropical dry forest- Disturbed | 30 °C; 70.9 %R.H.; 0 km/h Average wind Speed; 0 km/h max. wind Speed |
| *Culex (Melanoconion) erraticus* | Female | KM593022 | ZFMK-PR037 | COLOMBIA: Antioquia Dpto, La Pintada. N05.63622º W075.58782º. 659m. 24.ix. 2013, CDC. | Tropical dry forest- Disturbed | 30 °C; 70.9 %R.H.; 0 km/h Average wind Speed; 0 km/h max. wind Speed |
| *Culex (Melanoconion) erraticus* | Female | KM593023 | ZFMK-PR038 | COLOMBIA: Antioquia Dpto, La Pintada. N05.63622º W075.58782º. 659m. 24.ix. 2013, CDC. | Tropical dry forest- Disturbed | 30 °C; 70.9 %R.H.; 0 km/h Average wind Speed; 0 km/h max. wind Speed |
| *Culex (Melanoconion) erraticus* | Female | KM593024 | ZFMK-PR039 | COLOMBIA: Antioquia Dpto, La Pintada. N05.63622º W075.58782º. 659m. 24.ix. 2013, CDC. | Tropical dry forest- Disturbed | 30 °C; 70.9 %R.H.; 0 km/h Average wind Speed; 0 km/h max. wind Speed |
| *Culex (Melanoconion) erraticus* | Female | KM593029 | ZFMK-PR044 | COLOMBIA: Antioquia Dpto, La Pintada. N05.63622º W075.58782º. 659m. 25.ix. 2013, CDC. | Tropical dry forest- Disturbed | 37.2 °C; 47 %R.H.; 4.6 km/h Average wind Speed; 4.7 km/h max. wind Speed |
| *Culex (Melanoconion) erraticus* | Female | KM593031 | ZFMK-PR046 | COLOMBIA: Antioquia Dpto, La Pintada. N05.63622º W075.58782º. 659m. 25.ix. 2013, CDC. | Tropical dry forest- Disturbed | 37.2 °C; 47 %R.H.; 4.6 km/h Average wind Speed; 4.7 km/h max. wind Speed |
| *Culex (Melanoconion) erraticus* | Female | KM593032 | ZFMK-PR047 | COLOMBIA: Antioquia Dpto, La Pintada, N05.63646º W075.59002º. 710m. 25.ix. 2013, CDC. | Tropical dry forest- Forest | 28.3 °C; 94.8 %R.H.; 0 km/h Average wind Speed; 0 km/h max. wind Speed |
| *Culex (Melanoconion) erraticus* | Male | KM593041 | ZFMK-PR056 | COLOMBIA: Antioquia Dpto, La Pintada. N05.63622º W075.58782º. 659m. 24.ix. 2013, CDC. | Tropical dry forest- Disturbed | 30 °C; 70.9 %R.H.; 0 km/h Average wind Speed; 0 km/h max. wind Speed |
| *Culex (Melanoconion) erraticus* | Male | KM593045 | ZFMK-PR060 | COLOMBIA: Antioquia Dpto, La Pintada, N05.63646º W075.59002º. 710m. 24.ix. 2013, CDC. | Tropical dry forest- Forest | 27.3 °C; 80.4 %R.H.; 0 km/h Average wind Speed; 0 km/h max. wind Speed |
| *Culex (Melanoconion) lucifugus* | Female | KM593025 | ZFMK-PR040 | COLOMBIA: Antioquia Dpto, La Pintada, N05.63646º W075.59002º. 710m. 24.ix. 2013, CDC. | Tropical dry forest- Forest | 27.3 °C; 80.4 %R.H.; 0 km/h Average wind Speed; 0 km/h max. wind Speed |
| *Culex (Melanoconion) lucifugus* | Female | KM593027 | ZFMK-PR042 | COLOMBIA: Antioquia Dpto, La Pintada, N05.63646º W075.59002º. 710m. 25.ix. 2013, CDC. | Tropical dry forest- Forest | 28.3 °C; 94.8 %R.H.; 0 km/h Average wind Speed; 0 km/h max. wind Speed |
| *Culex (Melanoconion) lucifugus* | Male | KM593044 | ZFMK-PR059 | COLOMBIA: Antioquia Dpto, La Pintada, N05.63646º W075.59002º. 710m. 24.ix. 2013, CDC. | Tropical dry forest- Forest | 27.3 °C; 80.4 %R.H.; 0 km/h Average wind Speed; 0 km/h max. wind Speed |
| *Culex (Melanoconion) lucifugus* | Male | KM593047 | ZFMK-PR062 | COLOMBIA: Antioquia Dpto, La Pintada, N05.63646º W075.59002º. 710m. 24.ix. 2013, CDC. | Tropical dry forest- Forest | 27.3 °C; 80.4 %R.H.; 0 km/h Average wind Speed; 0 km/h max. wind Speed |
| *Culex (Melanoconion) lucifugus* | Male | KM593052 | ZFMK-PR067 | COLOMBIA: Antioquia Dpto, La Pintada, N05.63646º W075.59002º. 710m. 24.ix. 2013, CDC. | Tropical dry forest- Forest | 27.3 °C; 80.4 %R.H.; 0 km/h Average wind Speed; 0 km/h max. wind Speed |
| *Culex (Melanoconion) lucifugus* | Male | KM593056 | ZFMK-PR071 | COLOMBIA: Antioquia Dpto, La Pintada, N05.63646º W075.59002º. 710m. 24.ix. 2013, CDC. | Tropical dry forest- Forest | 27.3 °C; 80.4 %R.H.; 0 km/h Average wind Speed; 0 km/h max. wind Speed |
| *Culex (Melanoconion) theobaldi* | Male | KM593049 | ZFMK-PR064 | COLOMBIA: Antioquia Dpto, La Pintada, N05.63646º W075.59002º. 710m. 24.ix. 2013, CDC. | Tropical dry forest- Forest | 27.3 °C; 80.4 %R.H.; 0 km/h Average wind Speed; 0 km/h max. wind Speed |
| *Culex (Melanoconion)* sp. | Female | KM593020 | ZFMK-PR035 | COLOMBIA: Antioquia Dpto, La Pintada, N05.63646º W075.59002º. 710m. 24.ix. 2013, CDC. | Tropical dry forest- Forest | 27.3 °C; 80.4 %R.H.; 0 km/h Average wind Speed; 0 km/h max. wind Speed |
| *Culex (Melanoconion)* sp. | Female | KM593021 | ZFMK-PR036 | COLOMBIA: Antioquia Dpto, La Pintada, N05.63646º W075.59002º. 710m. 24.ix. 2013, CDC. | Tropical dry forest- Forest | 27.3 °C; 80.4 %R.H.; 0 km/h Average wind Speed; 0 km/h max. wind Speed |
| *Culex (Melanoconion)* sp. | Female | KM593026 | ZFMK-PR041 | COLOMBIA: Antioquia Dpto, La Pintada, N05.63646º W075.59002º. 710m. 25.ix. 2013, CDC. | Tropical dry forest- Forest | 28.3 °C; 94.8 %R.H.; 0 km/h Average wind Speed; 0 km/h max. wind Speed |
| *Culex (Melanoconion)* sp. | Female | KM593028 | ZFMK-PR043 | COLOMBIA: Antioquia Dpto, La Pintada, N05.63646º W075.59002º. 710m. 25.ix. 2013, CDC. | Tropical dry forest- Forest | 28.3 °C; 94.8 %R.H.; 0 km/h Average wind Speed; 0 km/h max. wind Speed |
| *Culex (Phenacomyia) corniger* | Female | KM592994 | ZFMK-PR009 | COLOMBIA: Caldas Dpto, Rio Sucio. N05.40523º W075.70572º. 1978m. 28.ix. 2013, CDC. | Cloud forest- Forest | 23.8 °C; 77.1 %R.H.; 0 km/h Average wind Speed; 0 km/h max. wind Speed |
| *Culex (Phenacomyia) corniger* | Female | KM592998 | ZFMK-PR013 | COLOMBIA: Caldas Dpto, Rio Sucio. N05.40523º W075.70572º 1978m. 27.ix. 2013, CDC. | Cloud forest- Forest | 19.3 °C; 89.4 %R.H.; 0 km/h Average wind Speed; 0 km/h max. wind Speed |
| *Culex (Phenacomyia) corniger* | Female | KM592999 | ZFMK-PR014 | COLOMBIA: Caldas Dpto, Rio Sucio. N05.40523º W075.70572º. 1978m. 27.ix. 2013, CDC. | Cloud forest- Forest | 19.3 °C; 89.4 %R.H.; 0 km/h Average wind Speed; 0 km/h max. wind Speed |
| *Culex (Phenacomyia) corniger* | Female | KM593000 | ZFMK-PR015 | COLOMBIA: Caldas Dpto, Rio Sucio. N05.40523º W075.70572º. 1978m. 28.ix. 2013, CDC. | Cloud forest- Forest | 23.8 °C; 77.1 %R.H.; 0 km/h Average wind Speed; 0 km/h max. wind Speed |
| *Culex (Phenacomyia) corniger* | Female | KM593002 | ZFMK-PR017 | COLOMBIA: Caldas Dpto, Rio Sucio. N05.40523º W075.70572º. 1978m. 27.ix. 2013, CDC. | Cloud forest- Forest | 19.3 °C; 89.4 %R.H.; 0 km/h Average wind Speed; 0 km/h max. wind Speed |
| *Culex (Phenacomyia) corniger* | Female | KM593003 | ZFMK-PR018 | COLOMBIA: Caldas Dpto, Rio Sucio. N05.40523º W075.70572º. 1978m. 27.ix. 2013, CDC. | Cloud forest- Forest | 19.3 °C; 89.4 %R.H.; 0 km/h Average wind Speed; 0 km/h max. wind Speed |
| *Culex (Phenacomyia) corniger* | Female | KM593004 | ZFMK-PR019 | COLOMBIA: Caldas Dpto, Rio Sucio. N05.40523º W075.70572º. 1978m. 27.ix. 2013, CDC. | Cloud forest- Forest | 19.3 °C; 89.4 %R.H.; 0 km/h Average wind Speed; 0 km/h max. wind Speed |
| *Culex (Phenacomyia) corniger* | Female | KM593005 | ZFMK-PR020 | COLOMBIA: Caldas Dpto, Rio Sucio. N05.40523º W075.70572º. 1978m. 27.ix. 2013, CDC. | Cloud forest- Forest | 19.3 °C; 89.4 %R.H.; 0 km/h Average wind Speed; 0 km/h max. wind Speed |
| *Culex (Phenacomyia) corniger* | Female | KM593006 | ZFMK-PR021 | COLOMBIA: Caldas Dpto, Rio Sucio. N05.40523º W075.70572º. 1978m. 28.ix. 2013, CDC. | Cloud forest- Forest | 23.8 °C; 77.1 %R.H.; 0 km/h Average wind Speed; 0 km/h max. wind Speed |
| *Culex (Phenacomyia) corniger* | Female | KM593015 | ZFMK-PR030 | COLOMBIA: Caldas Dpto, Rio Sucio. N05.40523º W075.70572º 1978m. 28.ix. 2013, CDC. | Cloud forest- Forest | 23.8 °C; 77.1 %R.H.; 0 km/h Average wind Speed; 0 km/h max. wind Speed |
| *Culex (Phenacomyia) lactator* | Female | KM592997 | ZFMK-PR012 | COLOMBIA: Antioquia Dpto, La Pintada, N05.63646º W075.59002º 710m. 24.ix. 2013, CDC. | Tropical dry forest- Forest | 27.3 °C; 80.4 %R.H.; 0 km/h Average wind Speed; 0 km/h max. wind Speed |
| *Culex (Phenacomyia) lactator* | Female | KM593001 | ZFMK-PR016 | COLOMBIA: Antioquia Dpto, La Pintada, N05.63646º W075.59002º. 710m. 25.ix. 2013, CDC. | Tropical dry forest- Forest | 28.3 °C; 94.8 %R.H.; 0 km/h Average wind Speed; 0 km/h max. wind Speed |
| *Culex (Phenacomyia) lactator* | Female | KM593012 | ZFMK-PR027 | COLOMBIA: Antioquia Dpto, La Pintada, N05.63646º W075.59002º. 710m. 25.ix. 2013, CDC. | Tropical dry forest- Forest | 28.3 °C; 94.8 %R.H.; 0 km/h Average wind Speed; 0 km/h max. wind Speed |
| *Haemagogus (Haemagogus)* c.f. *lucifer* | Female | KM593036 | ZFMK-PR051 | COLOMBIA: Antioquia Dpto, La Pintada, Hotel Pipinta. N05.63670º W075.59200º. 760m. 26.ix. 2013, Human Bait. | Tropical dry forest- Disturbed | 28;8 °C; 94;8 %R.H.; 0 km/h Average wind Speed; 0 km/h max. wind Speed |
| *Haemagogus (Haemagogus) janthinomys* | Female | KM593034 | ZFMK-PR049 | COLOMBIA: Antioquia Dpto, La Pintada, Hotel Pipinta. N05.63670º W075.59200º. 760m. 26.ix. 2013, Human Bait. | Tropical dry forest- Disturbed | 28;8 °C; 94;8 %R.H.; 0 km/h Average wind Speed; 0 km/h max. wind Speed |
| *Haemagogus (Haemagogus) janthinomys* | Female | KM593035 | ZFMK-PR050 | COLOMBIA: Antioquia Dpto, La Pintada, Hotel Pipinta. N05.63670º W075.59200º. 760m. 26.ix. 2013, Human Bait. | Tropical dry forest- Disturbed | 28;8 °C; 94;8 %R.H.; 0 km/h Average wind Speed; 0 km/h max. wind Speed |
| *Ochlerotatus euiris*  [uncertain subgenus] | Female | KM592988 | ZFMK-PR003 | COLOMBIA: Antioquia Dpto, Belmira. N06.64536º W075.67227º 3186m. 18.ix. 2013, Human Bait. | Paramo- Forest | 14.1 °C; 78.4 %R.H.; 1.2 km/h Average wind Speed; 8.4 km/h max. wind Speed |
| *Ochlerotatus (Ochlerotatus) angustivittatus* | Female | KM592987 | ZFMK-PR002 | COLOMBIA: Antioquia Dpto, La Pintada, N05.63646º W075.59002º 710m. 25.ix. 2013, Human Bait. | Tropical dry forest- Forest | 28.3 °C; 94.8 %R.H.; 0 km/h Average wind Speed; 0 km/h max. wind Speed |
| *Ochlerotatus (Ochlerotatus) angustivittatus* | Male | KM593061 | ZFMK-PR076 | COLOMBIA: Caldas Dpto, Supia. N5.43780º W75.64794º. 1150m. 28.ix. 2013. CDC | Rural area |  |
| *Psorophora (Grabhamia) cingulata* | Female | KM592989 | ZFMK-PR004 | COLOMBIA: Antioquia Dpto, La Pintada, N05.63646º W075.59002º. 710m. 24.ix. 2013, CDC. | Tropical dry forest- Disturbed | 30 °C; 70.9 %R.H.; 0 km/h Average wind Speed; 0 km/h max. wind Speed |
| *Psorophora (Janthinosoma) ferox* | Female | KM593037 | ZFMK-PR052 | COLOMBIA: Antioquia Dpto, La Pintada, N05.63646º W075.59002º. 710m. 25.ix. 2013, Human Bait. | Tropical dry forest- Forest | 28.3 °C; 94.8 %R.H.; 0 km/h Average wind Speed; 0 km/h max. wind Speed |
| *Psorophora (Janthinosoma) ferox* | Female | KM593038 | ZFMK-PR053 | COLOMBIA: Antioquia Dpto, La Pintada, N05.63646º W075.59002º 710m. 25.ix. 2013, Human Bait. | Tropical dry forest- Forest | 28.3 °C; 94.8 %R.H.; 0 km/h Average wind Speed; 0 km/h max. wind Speed |
| *Psorophora (Janthinosoma) ferox* | Male | KM593062 | ZFMK-PR077 | COLOMBIA: Antioquia Dpto, La Pintada. N05.63622º W075.58782º. 659m. 24- 26.ix. 2013,malaise. | Tropical dry forest- Disturbed |  |
| *Trichoprosopon evansae* | Female | KM593039 | ZFMK-PR054 | COLOMBIA: Caldas Dpto, Rio Sucio. N05.40523º W075.70572º. 1978m. 27.ix. 2013, Human Bait. | Cloud forest- Forest |  |
| *Wyeomyia (Dendromyia) luteoventralis* | Female | KM593040 | ZFMK-PR055 | COLOMBIA: Antioquia Dpto, La Pintada, N05.63646º W075.59002º 710m. 24.ix. 2013, CDC. | Tropical dry forest- Forest | 27.3 °C; 80.4 %R.H.; 0 km/h Average wind Speed; 0 km/h max. wind Speed |
| Unidentified | Female | KM592990 | ZFMK-PR005 | COLOMBIA: Caldas Dpto, Rio Sucio. N05.40523º W075.70572º 1978m. 27.ix. 2013, CDC. | Cloud forest- Forest | 19.3 °C; 89.4 %R.H.; 0 km/h Average wind Speed; 0 km/h max. wind Speed |
