# Supplementary material for: Mosquito species (Diptera, Culicidae) in three ecosystems from the Colombian Andes: identification through DNA barcoding and adult morphology
Source: Zookeys. 2015 Jul 15;(513):39–64. doi: 10.3897/zookeys.513.9561 (PMC4524277; doi:10.3897/zookeys.513.9561)
Supplement: Supplementary material 2 — COI mosquito sequences downloaded from NCBI and BOLD [file zookeys-513-039-s002.docx]

**APPENDIX 2**

Sequences from NCBI and BOLD. Sequences downloaded between December 2013 and February 2014 for all identified species with a minimum length of 480 bp of COI barcoding region, no stop codons and possible alignment among the majority of the sequences. Total of 1,159 sequences. In blue: Additional species sequences from other geographic areas for those Neotropical groups without available sequences from Neotropics.

| **Genus** | **Subgenus** | **Species** | **GenBank**  **Accession number** | **BOLD**  **Process ID** |
| --- | --- | --- | --- | --- |
| *Aedimorphus* |  | *vexans* |  | ACMC033-04;  ACMC104-04; ACMC198-04; ACMC221-04; ACMC276-04; ACMC277-04; ACMC288-04- ACMC290-04; CNPPC1722-12; CNPPE1804-12; CNPPE2056-12; CNPPE2225-12; CNPPF1245-12; GBDP14514-13; GBDP14515-13- GBDP14518-13; NEONT231-10- NEONT233-10; NEONT275-10; NEONT292-10; NEONT293-10; NEONU307-11; NEONU309-11; NEONU310-11; TDWGB123-10; TDWGB127-10; TDWGB129-10- TDWGB131-10; TDWGB133-10; TDWGB705-10 |
| *Anopheles* | *Anopheles* | *calderoni* |  | MBIB482-10; mBIB484-10; mBIB488-10;  mBIB489-10; mBIB490-10; mBIB491-10; mBIB680-10; mBIB681-10; mBIB682-10; mBIB688-10; mBIB694-10 |
| *Anopheles* | *Anopheles* | *costai* |  | GBANO479-12; GBANO480-12; GBANO797-12; GBANO798-12 |
| *Anopheles* | *Anopheles* | *intermedius* |  | GBDP12039-12 |
| *Anopheles* | *Anopheles* | *mattogrossensis* |  | GBANO796-12 |
| *Anopheles* | *Anopheles* | *neomaculipalpus* |  | GBANO794-12; GBANO795-12 |
| *Anopheles* | *Anopheles* | *peryassui* |  | GBANO481-12 |
| *Anopheles* | *Anopheles* | *pseudopunctipennis* |  | GBANO483-12; GBANO484-12; GBMIN14513-13; GBMIN14520-13 |
| *Anopheles* | *Anopheles* | *punctimacula* |  | GBANO792-12; GBMIN14514-13; mBIB013-10 |
| *Anopheles* | *Anopheles* | *quadrimaculatus* |  | ACMC061-04; ACMC062-04; ACMC097-04; ACMC098-04; ACMC118-04; ACMC134-04; ACMC174-04; ACMC175-04; ACMC176-04; ACMC178-04; ACMC244-04; ACMC300-04; GBDP2875-06 |
| *Anopheles* | *Kerteszia* | *cruzii* |  | GBDP12017-12 |
| *Anopheles* | *Kerteszia* | *homunculus* |  | GBANO753-12- GBANO762-12 |
| *Anopheles* | *Kerteszia* | *lepidotus* |  | MBII894-09 |
| *Anopheles* | *Kerteszia* | *pholidotus* |  | MBIE384-09; mBIE385-09 |
| *Anopheles* | *Nyssorhynchus* | *albertoi* |  | GBANO013-10- GBANO015-10 |
| *Anopheles* | *Nyssorhynchus* | *albimanus* |  | GBMIN14511-13; GBMIN14517-13; GBMIN14518-13 |
| *Anopheles* | *Nyssorhynchus* | *albitarsis* |  | ALBCO015-13 - ALBCO056-13; GBANO783-12- GBANO786-12; GBDP12005-12; GBDP13443-13; GBDP14617-13- GBDP14627-13; GBDP14650-13- GBDP14656-13; GBDP2012-06- GBDP2022-06; GBDP2038-06 - GBDP2041-06; GBMIN12948-13; GBMIN12963-13; mBIB870-10; mBIB871-10; mBIE022-09-mBIE050-09; mBIE068-09; mBIE078-09; mBIE161-09-mBIE168-09; mBIE170-09-mBIE173-09; mBIE178-09; mBIE297-09-mBIE299-09; mBIE333-09-mBIE337-09; mBIE351-09-mBIE360-09; mBIG107-09; mBIG115-09; mBII011-09; mBII012-09; mBII016-09; mBII017-09; mBII020-09-mBII023-09; mBII134-09; mBII135-09-mBII144-09; mBII147-09-mBII159-09; mBII161-09-mBII168-09; mBII171-09; mBII210-09-mBII213-09; mBII220-09-mBII228-09; mBII240-09-mBII262-09; mBII268-09; mBII390-09-mBII393-09; mBII394-09-mBII397-09; mBII400-09-mBII408-09; mBII412-09; mBII416-09-mBII418-09; mBII420-09-mBII422-09; mBII424-09; mBII439-09-mBII441-09; mBII463-09-mBII446-09; mBII466-09; mBII486-09; mBII488-09; mBII500-09; mBII837-09; mBII839-09; mBII853-09; mBII855-09-mBII859-09; mBII862-09; mBIK001-10-mBIK014-10; mBIK083-10; mBIK153-10; mBIK154-10; mBIK157-10; mBIK158-10 |
| *Anopheles* | *Nyssorhynchus* | *antunesi* |  | GBANO444-12- GBANO449-12; GBANO450-12 |
| *Anopheles* | *Nyssorhynchus* | *aquasalis* |  | GBMIN14512-13; GBMIN14519-13 |
| *Anopheles* | *Nyssorhynchus* | *argyritarsis* |  | GBANO471-12; GBANO471-12; GBDP12006-12- GBDP12010-12 |
| *Anopheles* | *Nyssorhynchus* | *arthuri* |  | GBANO016-10- GBANO021-10; GBMIN38721-13 - GBMIN38723-13; GBMIN38754-13- GBMIN38756-13 |
| *Anopheles* | *Nyssorhynchus* | *atacamensis* |  | GBDP12011-12 |
| *Anopheles* | *Nyssorhynchus* | *benarrochi* |  | GBANO767-12- GBANO780-12; GBDP12012-12- GBDP12014-12; GBDP12051-12; GBMIN11843-13- GBMIN11848-13 |
| *Anopheles* | *Nyssorhynchus* | *braziliensis* |  | GBANO767-12- GBANO780-12; GBDP12012-12- GBDP12014-13; GBDP2044-06; GBDP2045-06 |
| *Anopheles* | *Nyssorhynchus* | *darlingi* |  | GBANO767-12- GBANO780-12; GBDP12012-12- GBDP12014-14; GBDP2042-06; GBDP2043-06 ; GTENK038-11 |
| *Anopheles* | *Nyssorhynchus* | *deaneorum* |  | GBANO767-12- GBANO780-12; GBDP12012-12- GBDP12014-15; GBDP13442-13; GBDP2034-06- GBDP2037-06; GBMIN12962-13; mBII078-09-mBII087-09; mBII094-09; mBII097-09; mBII099-09; mBII135-09; mBII146-09; mBII425-09; mBII438-09; mBII595-09; mBII615-09 |
| *Anopheles* | *Nyssorhynchus* | *dunhami* |  | GBANO767-12- GBANO780-12; GBDP12012-12- GBDP12014-16 |
| *Anopheles* | *Nyssorhynchus* | *evansae* |  | GBANO767-12- GBANO780-12; GBDP12012-12- GBDP12014-17; GBMIN12946-13; GBMIN12947-13; GBMIN12960-13; GBMIN12961-13 |
| *Anopheles* | *Nyssorhynchus* | *galvaoi* |  | GBANO767-12- GBANO780-12; GBDP12012-12- GBDP12014-18; GBDP13157-13; GBMIN12945-13; GBMIN12959-13 |
| *Anopheles* | *Nyssorhynchus* | *goeldii* |  | GBANO767-12- GBANO780-12; GBDP12012-12- GBDP12014-19; GBMIN11361-13- GBMIN11377-13; GBMIN11400-13- GBMIN11410-13 |
| *Anopheles* | *Nyssorhynchus* | *guarani* |  | GBANO767-12- GBANO780-12; GBDP12012-12- GBDP12014-20 |
| *Anopheles* | *Nyssorhynchus* | *janconnae* |  | GBANO343-12- GBANO356-12; mBII301-09-mBII304-09; mBII340-09; mBII341-09; mBII346-09-mBII349-09; mBII351-09; mBII353-09; mBII357-09; mBII361-09; mBII367-09; mBII371-09-mBII374-09; mBII377-09; mBII760-09; mBII761-09; mBIK015-10-mBIK019-10; mBIK031-10-mBIK069-10; mBIK081-10-mBIK085-10; mBIK155-10 |
| *Anopheles* | *Nyssorhynchus* | *konderi* |  | GBANO637-12- GBANO646-12; GBDP12041-12 |
| *Anopheles* | *Nyssorhynchus* | *lanei* |  | GBDP12042-12; GBDP12043-12 |
| *Anopheles* | *Nyssorhynchus* | *lutzii* |  | GBDP11987-12- GBDP11995-12; GBMIN12940-13- GBMIN12944-13; GBMIN12955-13- GBMIN12958-13 |
| *Anopheles* | *Nyssorhynchus* | *marajoara* |  | GBANO476-12- GBANO478-12; GBDP12044-12; GBDP2024-06- GBDP2033-06; GBMIN12939-13; GBMIN12954-13; mBIE118-09-mBIE128-09; mBIE140-09-mBIE148-09; mBII025-09-mBII033-09; mBII060-09; mBII070-09; mBII072-09; mBII169-09; mBII170-09; mBII381-09; mBII385-09; mBII386-09; mBII499-09 |
| *Anopheles* | *Nyssorhynchus* | *nuneztovari* |  | GBANO605-12- GBANO614-12; GBDP12035-12- GBDP12038-12 |
| *Anopheles* | *Nyssorhynchus* | *oryzalimnetes* |  | GBDP12045-12; mBIE169-09; mBIE174-09; mBIE175-09; mBIE177-09; mBIE179-09; mBIE180-09; mBIE181-09; mBIE185-09-mBIE187-09; mBII010-09; mBII014-09; mBII102-09; mBII103-09; mBII106-09; mBII108-09; mBII110-09; mBII114-09; mBII1021-09-mBII124-09; mBII1028-09-mBII1033-09; mBII160-09; mBII173-09; mBII177-09; mBII178-09; mBII206-09 -mBII209-09; mBII295-09; mBII296-09; mBII393-09; mBII413-09; mBII471-09; mBII485-09; mBII489-09; mBII507-09; mBII510-09 |
| *Anopheles* | *Nyssorhynchus* | *oswaldoi* |  | GBANO603-12; GBDP12046-12- GBDP12049-12; GBMIN12937-13; GBMIN12938-13; GBMIN12952-13; GBMIN12953-13 |
| *Anopheles* | *Nyssorhynchus* | *parvus* |  | GBDP11996-12- GBDP12004-12 |
| *Anopheles* | *Nyssorhynchus* | *pristinus* |  | GBANO450-12; GBANO452-12- GBANO461-12 |
| *Anopheles* | *Nyssorhynchus* | *rangeli* |  | GBANO466-12- GBANO470-12; GBANO602-12; GBANO787-12- GBANO791-12; GBDP12050-12; GBMIN12936-13; GBMIN12951-13 |
| *Anopheles* | *Nyssorhynchus* | *rondoni* |  | GBDP12052-12- GBDP12054-12 |
| *Anopheles* | *Nyssorhynchus* | *strodei* |  | GBANO001-10- GBANO012-10; GBANO022-10; GBDP12055-12; GBDP12056-12; GBDP13178-13- GBDP13189-13; GBMIN12935-13; GBMIN12948-13; GBMIN12949-13; GBMIN12950-13 |
| *Anopheles* | *Nyssorhynchus* | *triannulatus* |  | GBANO463-12 - GBANO465-12; GBANO781-12- GBANO783-12; GBDP12057-12- GBDP12061-12; GBMIN11388-13- GBMIN11399-13; GBMIN11417-13- GBMIN11430-13 |
| *Anopheles* | *Nyssorhynchus* | *trinkae* |  | GBANO619-12; GBANO620-12 |
| *Anopheles* | *Stethomyia* | *kompi* |  | GBDP12040-12 |
| *Anopheles* | *Stethomyia* | *nimbus* |  | GBANO485-12 |
| *Coquillettidia* | *Coquillettidia* | *aurea* | GQ165803 |  |
| *Coquillettidia* | *Coquillettidia* | *aurites* | GQ165802 |  |
| *Coquillettidia* | *Coquillettidia* | *crassipes* | JQ728121- JQ728125; JQ728179; JQ728319 |  |
| *Coquillettidia* | *Coquillettidia* | *fuscopennata* | GQ165802 |  |
| *Coquillettidia* | *Coquillettidia* | *maculipennis* | GQ165785 |  |
| *Coquillettidia* | *Coquillettidia* | *metallica* | GQ165789 |  |
| *Coquillettidia* | *Coquillettidia* | *perturbans* | KF761600; GU908055; GU908066- GU908073; JX259890- JX259897; GU013591 |  |
| *Coquillettidia* | *Coquillettidia* | *richiardii* | JQ728309; JQ728310; JX040513 |  |
| *Culex* | *Culex* | *apicinus* | HE600031 |  |
| *Culex* | *Culex* | *brethesi* |  | GBDCU312-12 |
| *Culex* | *Culex* | *chidesteri* | HE600028 |  |
| *Culex* | *Culex* | *dolosus* | HE600027 |  |
| *Culex* | *Culex* | *eduardoi* | HE605120 |  |
| *Culex* | *Culex* | *interrogator* |  | NEONV034-11 |
| *Culex* | *Culex* | *maxi* | HE599225 |  |
| *Culex* | *Culex* | *nigripalpus* |  | NEONV041-11 |
| *Culex* | *Culex* | *quinquefasciatus* |  | GBDCU221-12- GBDCU228-12; GBDCU237-12; GBDCU361-12; GBDCU639-12-GBDCU655-12; GBDP12712-12; GBDP13297-13; GBDP13304-13- GBDP13306-13; GBDP13331-13; GBDP13332-13; GBDP13371-13; GBDP13372-13; GBDP13406-13; GBDP13410-13; GBDP13484-13; GBDP13552-13; GBDP4840-08; GBDP5691-09; GBDP5692-09; GBDP5700-09; GBDP7990-09; GBDP7991-09; GBDP7993-09; GBDP7994-09; GTENK034-11; AMTB1058-12 |
| *Culex* | *Culex* | *restuans* |  | ACMC051-04; ACMC106-04; ACMC245-04-ACMC254-04; CNPPB1553-12; NEONU143-11; NEONU144-11; NEONU146-11- NEONU148-11 |
| *Culex* | *Culex* | *spinosus* | KF919191 |  |
| *Culex* | *Melanoconion* | *bahiensis* |  | NEONV018-11 |
| *Culex* | *Melanoconion* | *inhibitator* |  | NEONV008-11 |
| *Culex* | *Neoculex* | *apicalis* | JX259906; JX259905 |  |
| *Culex* | *Neoculex* | *territans* | JX259921- JX259933; GU908105; GU908104 |  |
| *Culex* | *Phytotelmatomyia* | *renatoi* | HE605119 |  |
| *Gymnometopa* |  | *mediovittata* |  | NEONV143-11 |
| *Howardina* |  | *bahamensis* |  | NEONV117-11; NEONV118-11 |
| *Hulecoeteomyia* |  | *japonica* |  | GBDCU002-12; GBDCU609-12; GBDP8040-09- GBDP8048-09; NEONU116-11- NEONU123-11; NEONU185-11; TDWGB124-10; TDWGB126-10 |
| *Lewnielsenius* |  | *muelleri* |  | NEONV157-11 |
| *Mansonia* | *Mansonia* | *flaveola* | JX260065 | NEONV060-11 |
| *Mansonia* | *Mansonioides* | *annulata* | HQ341634- HQ341642 |  |
| *Mansonia* | *Mansonioides* | *dives* | JF811421- JF811428 |  |
| *Ochlerotatus* | *Culicelsa* | *sollicitans* |  | ACMC260-04- ACMC264-04; TDWGB126-10 |
| *Ochlerotatus* | *Culicelsa* | *taeniorhynchus* |  | NEONV180-11 |
| *Ochlerotatus* | *Ochlerotatus* | *crinifer* | HE605118 |  |
| *Ochlerotatus* | *Ochlerotatus* | *tortilis* |  | NEONV182-11- NEONV185-11 |
| *Ochlerotatus* | subgenus uncertain | *albifasciatus* | HE599223 |  |
| *Ochlerotatus* | *Protoculex* | *atlanticus* | JX259518; JX259519 |  |
| *Orthopodomyia* |  | *alba* | GU908120 |  |
| *Orthopodomyia* |  | *anopheloides* | AY917200; JQ728140- JQ728138 |  |
| *Psorophora* | *Grabhamia* | *columbiae* |  | NEONV064-11; NEONV065-11 |
| *Psorophora* | *Grabhamia* | *insularia* |  | NEONV070-11; NEONV071-11 |
| *Psorophora* | *Grabhamia* | *pygmaea* |  | NEONV077-11; NEONV078-11 |
| *Psorophora* | *Janthinosoma* | *cyanescens* | HE599224 |  |
| *Psorophora* | *Psorophora* | *howardii* |  | NEONV083-11 |
| *Sabethes* | *Sabethes* | *cyaneus* |  | ACMC003-04 |
| *Stegomyia* | *Stegomyia* | *aegypti* |  | ACMC007-04; GBDCU236-12; GBDCU308-12- GBDCU311-12; GBDP0799-06; GBDP0799-06- GBDP0808-06- GBDP0815-06; GBDP10706-12; GBDP4310-07; GBDP6753-09- GBDP6755-09; GTENK039-11 |
| *Stegomyia* | subgenus uncertain | *albopicta* |  | ACLB004-06; GBDCU378-12; GBDCU379-12; GBDCU414-12; GBDCU536-12- GBDCU556-12; GBDCU608-12; GBDCU672-12- GBDCU685-12; GBDP0816-06- GBDP0827-06; GBDP10707-12; GBDP12091-12- GBDP12097-12; GBDP12346-12; GBDP12347-12; GBDP12351-12; GBDP13300-13; GBDP13302-13; GBDP13309-13; GBDP13316-13; GBDP13369-13; GBDP13370-13; GBDP13399-13; GBDP13407-13; GBDP13418-13; GBDP13431-13; GBDP13481-13; GBDP13497-13; GBDP13516-13; GBDP13517-13; GBDP13548-13; GBDP14272-13; GBDP14273-13; GBDP14335-13 - GBDP14337-13; BIT1101-11; BIT1102-11; BIT1104-11 |
| *Toxorhynchites* | *Lynchiella* | *rutilus* | GU908124; AF425849 |  |
| *Toxorhynchites* | *Toxorhynchites* | *christophi* | JQ728204 |  |
| *Toxorhynchites* | *Toxorhynchites* | *edwardsi* | JQ728337 |  |
| *Toxorhynchites* | *Toxorhynchites* | *gravelyi* | JQ728330; JQ728210; JQ728144 |  |
| *Toxorhynchites* | *Toxorhynchites* | *kempi* | JQ728329 |  |
| *Toxorhynchites* | *Toxorhynchites* | *splendens* | JQ728340; JQ728127; JQ728126; HQ398877 |  |
| *Uranotaenia* | *Uranotaenia* | *lowii* | HE600029 |  |
| *Uranotaenia* | *Uranotaenia* | *sapphirina* |  | ACMC076-004; ACMC080-004; ACMC243-04; ACMC271-04; ACMC272-04 |
| *Uranotaenia* | *Uranotaenia* | *socialis* |  | NEONV287-11 |
