# Supplementary material for: Mosquito species (Diptera, Culicidae) in three ecosystems from the Colombian Andes: identification through DNA barcoding and adult morphology
Source: Zookeys. 2015 Jul 15;(513):39–64. doi: 10.3897/zookeys.513.9561 (PMC4524277; doi:10.3897/zookeys.513.9561)
Supplement: Supplementary material 3 — Mosquito specimens collected in Uraba during 2009 (unpublish data) [file zookeys-513-039-s003.docx]

**APPENDIX 3**

Additional sequences of mosquitoes collected in rural areas of Uraba (Antioquia, Colombia) during 2009 including sampling information.

| **Species** | **Gender** | **Code** | **Label information** |
| --- | --- | --- | --- |
|  |  |  |  |
| *Anopheles (Anopheles) neomaculipalpus* | Female | 30AN | COLOMBIA: Antioquia Dpto, Turbo. N7.913889º W76.6025º 185m., 13.ii.2009, CDC. |
| *Anopheles (Nyssorhynchus) nuneztovari* |  | 463AN | COLOMBIA: Antioquia Dpto, Apartado. N7.893889º W76.595833º 83m., 09.xi.2009, CDC. |
| *Anopheles (Nyssorhynchus) nuneztovari* |  | 466AN | COLOMBIA: Antioquia Dpto, Turbo. N7.913889º W76.6025º 185m., 13.ii.2009, Human Bait. |
| *Anopheles (Nyssorhynchus) oswaldoi B* |  | 465AN | COLOMBIA: Antioquia Dpto, Turbo. N7.913889º W76.6025º 185m., 13.ii.2009, CDC. |
| *Coquillettidia (Rhyncotaenia) venezuelensis* | Female | 601CQ | COLOMBIA: Antioquia Dpto, Turbo. N7.913889º W76.6025º 185m., 13.ii.2009, Human Bait. |
| *Coquillettidia (Rhyncotaenia) venezuelensis* | Female | 602CQ | COLOMBIA: Antioquia Dpto, Turbo. N7.913889º W76.6025º 185m., 13.ii.2009, Human Bait. |
| *Coquillettidia (Rhyncotaenia) venezuelensis* | Female | 607CQ | COLOMBIA: Antioquia Dpto, Turbo. N7.913889º W76.6025º 185m., 13.ii.2009, Human Bait. |
| *Culex (Culex) declarator* | Male | 829CX | COLOMBIA: Antioquia Dpto, Turbo. N7.913889º W76.6025º 185m., 14.iv.2009, CDC. |
| *Culex (Culex) nigripalpus* |  | 512CX | COLOMBIA: Antioquia Dpto, Turbo. N7.913889º W76.6025º 185m., 13.ii.2009, CDC. |
| *Culex (Culex) nigripalpus* |  | 513CX | COLOMBIA: Antioquia Dpto, Turbo. N7.913889º W76.6025º 185m., 13.ii.2009, CDC. |
| *Culex (Culex) nigripalpus* | Female | 671CX | COLOMBIA: Antioquia Dpto, Turbo. N7.913889º W76.6025º 185m., 14.iv.2009, CDC. |
| *Culex (Culex) nigripalpus* | Male | 677CX | COLOMBIA: Antioquia Dpto, Turbo. N7.913889º W76.6025º 185m., 14.iv.2009, CDC. |
| *Culex (Culex) nigripalpus* |  | 678CX | COLOMBIA: Antioquia Dpto, Turbo. N7.913889º W76.6025º 185m., 14.iv.2009, CDC. |
| *Culex (Culex) nigripalpus* |  | 696CX | COLOMBIA: Antioquia Dpto, Turbo. N7.913889º W76.6025º 185m., 14.iv.2009, CDC. |
| *Culex (Culex) nigripalpus* |  | 705CX | COLOMBIA: Antioquia Dpto, Turbo. N7.913889º W76.6025º 185m., 14.iv.2009, CDC. |
| *Culex (Culex) nigripalpus* |  | 7CX | COLOMBIA: Antioquia Dpto, Turbo. N7.913889º W76.6025º 185m., 14.iv.2009, CDC. |
| *Culex (Culex) nigripalpus* |  | 856CX | COLOMBIA: Antioquia Dpto, Turbo. N7.913889º W76.6025º 185m., 13.ii.2009, CDC. |
| *Culex (Culex) quinquefasciatus* | Male | 869CX | COLOMBIA: Antioquia Dpto, Apartado. N7.893889º W76.595833º 83m., 09.xi.2009, CDC. |
| *Culex (Culex) quinquefasciatus* | Male | 872CX | COLOMBIA: Antioquia Dpto, Apartado. N8.069444º W76.588056º 116m., 09.xi.2009, CDC. |
| *Culex (Culex) quinquefasciatus* | Male | 876CX | COLOMBIA: Antioquia Dpto, Apartado. N7.893889º W76.595833º 83m., 09.xi.2009, CDC. |
| *Culex* sp. |  | 29CX | COLOMBIA: Antioquia Dpto, Turbo. N7.913889º W76.6025º 185m., 13.ii.2009, CDC. |
| *Culex* sp. |  | 505CX | COLOMBIA: Antioquia Dpto, Turbo. N7.913889º W76.6025º 185m., 13.ii.2009, CDC. |
| *Culex* sp. |  | 6CX | COLOMBIA: Antioquia Dpto, Turbo. N7.913889º W76.6025º 185m., 13.ii.2009, CDC. |
| *Isostomyia espini* | Female | 574IS | COLOMBIA: Antioquia Dpto, Turbo. N7.913889º W76.6025º 185m., 13.ii.2009, Human Bait. |
| *Johnbelkinia leucopus* | Female | 575JO | COLOMBIA: Antioquia Dpto, Turbo. N7.913889º W76.6025º 185m., 13.ii.2009, Human Bait. |
| *Johnbelkinia leucopus* | Female | 854JO | COLOMBIA: Antioquia Dpto, Turbo. N7.913889º W76.6025º 185m., 13.ii.2009, Human Bait. |
| *Mansonia (Mansonia) pseudotitillans* | Female | 479MA | COLOMBIA: Antioquia Dpto, Apartado. N7.893889º W76.595833º 83m., 10.xi.2009, Shannon. |
| *Ochlerotatus (Ochlerotatus) scapularis* | Female | 535AE | COLOMBIA: Antioquia Dpto, Turbo. N7.913889º W76.6025º 185m., 13.ii.2009, Human Bait. |
| *Ochlerotatus (Ochlerotatus) scapularis* |  | 539AE | COLOMBIA: Antioquia Dpto, Turbo. N7.913889º W76.6025º 185m., 13.ii.2009, Human Bait. |
| *Ochlerotatus (Ochlerotatus) scapularis* | Female | 604AE | COLOMBIA: Antioquia Dpto, Turbo. N7.913889º W76.6025º 185m., 13.ii.2009, Human Bait. |
| *Ochlerotatus (Protoculex) serratus* |  | 481AE | COLOMBIA: Antioquia Dpto, Apartado. N7.893889º W76.595833º 83m.,10.xi.2009, Shannon. |
| *Ochlerotatus (Protoculex) serratus* |  | 8AE | COLOMBIA: Antioquia Dpto, Turbo. N7.913889º W76.6025º 185m., 14.iv.2009, CDC. |
| *Ochlerotatus* sp. |  | 0831AE | COLOMBIA: Antioquia Dpto, Turbo. N7.913889º W76.6025º 185m., 14.iv.2009, CDC. |
| *Ochlerotatus* sp. | Female | 557AE | COLOMBIA: Antioquia Dpto, Turbo. N7.913889º W76.6025º 185m., 13.ii.2009, Human Bait. |
| *Psorophora (Janthinosoma) ferox* | Female | 531PS | COLOMBIA: Antioquia Dpto, Turbo. N7.913889º W76.6025º 185m., 13.ii.2009, Human Bait. |
| *Psorophora (Janthinosoma) ferox* | Female | 573PS | COLOMBIA: Antioquia Dpto, Turbo. N7.913889º W76.6025º 185m., 14.iv.2009, Human Bait. |
| *Psorophora (Janthinosoma) ferox* | Female | 594PS | COLOMBIA: Antioquia Dpto, Turbo. N7.913889º W76.6025º 185m., 13.ii.2009, Human Bait. |
| *Psorophora (Psorophora) saeva* | Female | 587PS | COLOMBIA: Antioquia Dpto, Turbo. N7.913889º W76.6025º 185m., 13.ii.2009, Human Bait. |
| *Trichoprosopon compresum* | Female | 758TR | COLOMBIA: Antioquia Dpto, Apartado. N7.893889º W76.595833º 83m., 13.iv.2009, Human Bait. |
| *Trichoprosopon digitatum* | Female | 16TR | COLOMBIA: Antioquia Dpto, Apartado. N8.069444º W76.588056º 94m., 08.xi.2009, Human Bait. |
| *Trichoprosopon digitatum* | Female | 489TR | COLOMBIA: Antioquia Dpto, Apartado. N8.069444º W76.588056º 94m., 08.xi.2009, Human Bait. |
| *Trichoprosopon digitatum* | Female | 490TR | COLOMBIA: Antioquia Dpto, Apartado. N8.069444º W76.588056º 94m., 08.xi.2009, Human Bait. |
| *Trichoprosopon digitatum* | Female | 756TR | COLOMBIA: Antioquia Dpto, Apartado. N7.893889º W76.595833º 83m., 10.xi.2009, CDC. |
| *Uranotaenia (Uranotaenia) calosomata* | Male | 2UR | COLOMBIA: Antioquia Dpto, Apartado. N7.893889º W76.595833º 83m.,10.xi.2009, CDC. |
| *Uranotaenia* sp. | Male | 890UR | COLOMBIA: Antioquia Dpto, Apartado. N8.069444º W76.588056º 116m., 10.xi.2009, Human Bait. |
