# Supplementary material for: Mosquito species (Diptera, Culicidae) in three ecosystems from the Colombian Andes: identification through DNA barcoding and adult morphology
Source: Zookeys. 2015 Jul 15;(513):39–64. doi: 10.3897/zookeys.513.9561 (PMC4524277; doi:10.3897/zookeys.513.9561)
Supplement: Supplementary material 4 — Outgroup taxa used in the present study [file zookeys-513-039-s004.docx]

**APPENDIX 4**

Outgroup taxa used in the present study. Sequences downloaded from BOLD between December 2013 and February 2014.

| **Family** | **Genus** | **species** | **BOLD Process IDs** |
| --- | --- | --- | --- |
| Chironomidae | *Cricotopus* | *bicinctus* | CFWIH107-10 |
| Chironomidae | *Chironomus* | *decorus* | CNPPB139-12 |
| Chironomidae | *Chironomus* | *kiiensis* | GBDP12438-12 |
| Chironomidae | *Dicrotendipes* | *tritomus* | CNSLJ334-12 |
| Chironomidae | *Tanytarsus* | *guerlus* | CNSLJ343-12 |
| Dixidae | *Dixella* | sp. | SJAD6020-13 |
| Psychodidae | *Lutzomyia* | *longipalpis* | GBMIN23018-13 |
| Simuliidae | *Simulium* | *inaequale* | NWBF604-11 |
| Simuliidae | *Simulium* | *ochraceum* | NHMBF035-11 |
| Simuliidae | *Gigantodax* | *abalosi* | WBF641-11 |
| Simuliidae | *Gigantodax* | *basinflatus* | NWBF653-11 |
