# Supplementary material for: Mosquito species (Diptera, Culicidae) in three ecosystems from the Colombian Andes: identification through DNA barcoding and adult morphology
Source: Zookeys. 2015 Jul 15;(513):39–64. doi: 10.3897/zookeys.513.9561 (PMC4524277; doi:10.3897/zookeys.513.9561)

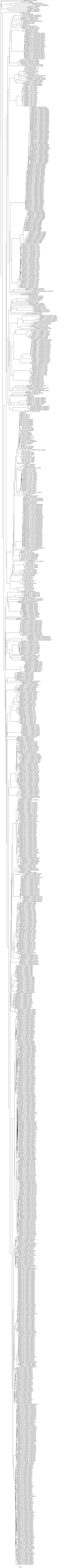

Supplement: Supplementary material 5 — NJ tree based on 1,292 sequences of Neotropical mosquitoes [file zookeys-513-039-s005.pdf]
