# Supplementary material for: Mosquito species (Diptera, Culicidae) in three ecosystems from the Colombian Andes: identification through DNA barcoding and adult morphology
Source: Zookeys. 2015 Jul 15;(513):39–64. doi: 10.3897/zookeys.513.9561 (PMC4524277; doi:10.3897/zookeys.513.9561)
Supplement: Supplementary material 7 — ML tree based on 1,292 sequences of Neotropical mosquitoes [file zookeys-513-039-s007.pdf]

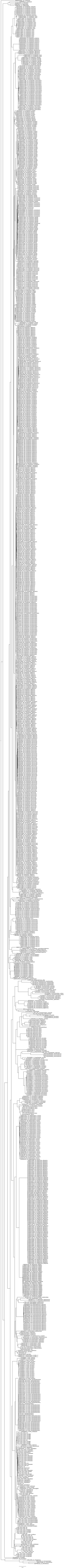
876Culex_quinquefasciatus
872Culex_quinquefasciatus
GBDP13306_13_Culex_quinquefasciatus
GBDP13305_13_Culex_quinquefasciatus
GBDP13331_13_Culex_quinquefasciatus
GBDP13332_13_Culex_quinquefasciatus
GBDP13552_13_Culex_quinquefasciatus
GBDCU221_12_Culex_quinquefasciatus
GBDP4840_08_Culex_quinquefasciatus
GBDP7994_09_Culex_quinquefasciatus
GBDP7993_09_Culex_quinquefasciatus
GBDP7990_09_Culex_quinquefasciatus
GBDP5691_09_Culex_quinquefasciatus
GTENK034_11_Culex_quinquefasciatus
GBDCU361_12_Culex_quinquefasciatus
HE600028.1_Culex_chidesteri
P13_COI_Culex_sp
P17_COI_Culex_corniger
P18_COI_Culex_corniger
P20_COI_Culex_corniger
P15_COI_Culex_corniger
P19_COI_Culex_corniger
P09_COI_Culex_sp
P30_COI_Culex_corniger
P21_COI_Culex_corniger
P14_COI_Culex_corniger
HE600031.1_Culex_apicinus
NEONV018_11_Culex_bahamensis
P73_COI_Culex_nigripalpus
NEONV041_11_Culex_nigripalpus
P69_COI_Culex_conspirator
P23_COI_Culex_nigripalpus
513Culex_nigripalpus1
P63_COI_Culex_conspirator
P70_COI_Culex_sp
P66_COI_Culex_declarator
P34_COI_Culex_declarator
P29_COI_Culex_declarator
P32_COI_Culex_declarator
P72_COI_Culex_declarator
505Culex_sp
P10_COI_Culex_sp
P61_COI_Culex_declarator
P06_COI_Culex_sp
29Culex_sp
829Culex_declarator
P08_COI_Culex_sp
P22_COI_Culex_declarator
P16_COI_Culex_lactator
P12_COI_Culex_sp
P27_COI_Culex_lactator
HE600027.1_Culex_dolosus
P25_COI_Culex_declarator
7Culex_nigripalpus
P07_COI_Culex_nigripalpus
856Culex_nigripalpus
696Culex_nigripalpus1
671Culex_nigripalpus
6Culex_sp
677Culex_nigripalpus
KF919191.1_Culex_spinosus
HE605119.1_Culex_renatoi
NEONV034_11_Culex_interrogator
678Culex_nigriplapus2
512Culex_nigripalpus2
705Culex_nigripalpus
P74_COI_Culex_spinosus
P75_COI_Culex_sp
ACMC247_04_Culex_restuans
ACMC248_04_Culex_restuans
ACMC250_04_Culex_restuans
ACMC106_04_Culex_restuans
NEONU143_11_Culex_restuans
NEONU148_11_Culex_restuans
ACMC246_04_Culex_restuans
ACMC253_04_Culex_restuans
NEONU147_11_Culex_restuans
NEONU144_11_Culex_restuans
NEONU146_11_Culex_restuans
ACMC051_04_Culex_restuans
ACMC249_04_Culex_restuans
ACMC254_04_Culex_restuans
ACMC251_04_Culex_restuans
ACMC252_04_Culex_restuans
ACMC245_04_Culex_restuans
CNPPB1553_12_Culex_restuans
P26_COI_Culex_cf_erythrothorax
P28_COI_Culex_CoronatorComplex
P33_COI_Culex_CoronatorComplex
P11_COI_Culex_CoronatorComplex
P31_COI_Culex_CoronatorComplex
HE599225.1_Culex_maxi
HE605120.1_Culex_eduardoi
P24_COI_Culex_CoronatorComplex
P40_COI_Culex_sp
P59_COI_Culex_sp
P62_COI_culex_lucifugus
P71_COI_Culex_lucifugus
P67_COI_Culex_lucifugus
P42_COI_Culex_sp
P60_COI_Culex_erraticus
P38_COI_Culex_sp
P37_COI_Culex_sp
P44_COI_Culex_sp
P56_COI_Culex_erraticus
P39_COI_Culex_sp
P47_COI_Culex_sp
P46_COI_Culex_sp
NEONV008_11_Culex_inhibitator
P64_COI_Culex_theobaldi
P45_COI_Culex_cf_spissipes
P36_COI_Culex_sp
P41_COI_Culex_sp
P35_COI_Culex_sp
P43_COI_Culex_sp
P57_COI_Culex_educator
P65_COI_Culex_conspirator
P58_COI_Culex_conspirator
P68_COI_Culex_conspirator
SSJAD6020_13_Dixella
CFWIH107_10_Cricotopus
CNSLJ343_12_Tanytarsus
CNSLJ334_12_Dicrotendipes
GBDP12438_12_Chironomus
CNPPB139_12_Chironomus
0.2
